# Terminology in ecology and evolutionary biology disproportionately harms marginalized groups

**DOI:** 10.1371/journal.pbio.3002933

**Published:** 2025-01-06

**Authors:** Mallory M. Rice, Shersingh Joseph Tumber-Dávila, Marcella D. Baiz, Susan J. Cheng, Kathy Darragh, Cesar O. Estien, J. W. Hammond, Danielle D. Ignace, Lily Khadempour, Kaitlyn M. Gaynor, Kirby L. Mills, Justine A. Smith, Alex C. Moore

**Affiliations:** 1 Department of Biological Sciences, California State University San Marcos, San Marcos, California, United States of America; 2 Department of Environmental Studies, Dartmouth College, Hanover, New Hampshire, United States of America; 3 Department of Biological Sciences, University at Buffalo, Buffalo, New York, United States of America; 4 Department of Mechanical Engineering and Department of Ecology and Evolutionary Biology, University of Michigan, Ann Arbor, Michigan, United States of America; 5 Department of Biology, Indiana University, Bloomington, Indiana, United States of America; 6 Department of Environmental Science, Policy, and Management, University of California, Berkeley, Berkeley, California, United States of America; 7 Department of Humanities, Michigan Technological University, Houghton, Michigan, United States of America; 8 Department of Forest and Conservation Sciences, University of British Columbia, Vancouver, Canada; 9 Department of Earth & Environmental Sciences, Rutgers University Newark, Newark, New Jersey, United States of America; 10 Department of Zoology, The University of British Columbia, University of British Columbia, Vancouver, Canada; 11 Institute for Global Change Biology, University of Michigan, Ann Arbor, Michigan, United States of America; 12 Department of Wildlife, Fish, and Conservation Biology, University of California, Davis, Davis, California, United States of America; 13 Department of Forest and Conservation Sciences, Department of Botany, University of British Columbia, Vancouver, Canada; The University of Queensland, AUSTRALIA

## Abstract

The discipline of ecology and evolutionary biology (EEB) has long grappled with issues of inclusivity and representation, particularly for individuals with systematically excluded and marginalized backgrounds or identities. For example, significant representation disparities still persist that disproportionately affect women and gender minorities; Black, Indigenous, and People of Color (BIPOC); individuals with disabilities; and people who are LGBTQIA+. Recent calls for action have urged the EEB community to directly address issues of representation, inclusion, justice, and equity. One aspect of this endeavor is to examine the use of EEB’s discipline-specific language and terminology, which may have the potential to perpetuate unjust systems and isolate marginalized groups. Through a mixed-methods survey, we examined how members of the EEB community perceive discipline-specific terminology, including how they believe it can be harmful and which terms they identified as problematic. Of the 795 survey respondents, we found that almost half agreed that there are harmful terms in EEB and that many individuals from marginalized groups responded that they have been harmed by such terminology. Most of the terms identified as harmful relate to race, ethnicity, and immigration; sex and gender; geopolitical hierarchies; and historical violence. Our findings suggest there is an urgent need for EEB to confront and critically reassess its discipline-specific terminology. By identifying harmful terms and their impacts, our study represents a crucial first step toward dismantling deeply rooted exclusionary structures in EEB. We encourage individuals, communities, and institutions to use these findings to reevaluate language used in disciplinary research, teaching and mentoring, manuscripts, and professional societies. Rectifying current harms in EEB will help promote a more just and inclusive discipline.

## Abstract 2

The discipline of ecology and evolutionary biology has long grappled with issues of inclusivity and representation. This study finds that individuals from marginalized groups were more likely to be harmed by terminology in ecology and evolutionary biology, and found over 200 discipline-specific terms were perceived by participants as harmful.

## Introduction

The discipline of ecology and evolutionary biology (EEB) has consistently struggled to recruit, retain, recognize, and create equitable environments for scholars from systematically excluded and marginalized backgrounds [1–7]. Experiences of exclusion and inequity are especially true for women and gender minorities; Black, Indigenous, and People of Color (BIPOC); those with disabilities; and people who are lesbian, gay, bisexual, transgender, queer/questioning, intersex, and asexual (LGBTQIA+). For example, the most recent Diversity and STEM report from the National Center for Science and Engineering Statistics found that as of 2021, individuals who identify as Black or African American made up only 6% of the total enrollment in United States (US) graduate programs in agricultural and veterinary sciences, biological and biomedical sciences, and natural resources and conservation [6], despite representing 14% of the US population [8]. Despite these low enrollments, graduate rates for these individuals are likely to be even lower due to financial barriers, discrimination, and a lack of institutional support [9,10]. Additionally, individuals with disabilities comprised roughly 10% of all doctorate degree recipients in these fields in 2021 [6] despite making up 27% of the adult population in the US by some estimates [11]. Mechanisms that contribute to these patterns of exclusion include disparities in financial resources and employment opportunities, limited mentorship, and experiences of implicit bias, isolation, harassment, and discrimination (e.g., [12–20]). Such mechanisms disproportionately harm marginalized individuals and limit the capacity for disciplinary growth by building knowledge on a narrow set of perspectives and research interests (e.g., [21–26]).

In recent years, there have been numerous calls for the EEB community to directly advocate for and engage in actions that increase representation within the discipline (e.g., [4,5,26–30]). For example, Cronin and colleagues [29] developed an evidence-based “toolkit” of interventions that promote anti-racism in ecology, evolution, and conservation classrooms, research laboratories, and departments. These interventions include highlighting research by members of systemically excluded groups, establishing a diverse network of mentors for students within and across lab groups, and adding land acknowledgments to presentations and publications [29]. Similarly, Chaudhury and Colla [28] highlight steps that can be taken to dismantle barriers to entry and success in ecology and conservation, such as direct engagement with anti-oppressive practices and applying an intersectional lens to diversity, equity, and inclusion efforts.

Among the various approaches that have been suggested to address the harms and barriers faced by systemically excluded and marginalized groups in science, calls to critically (re) examine discipline-specific language and terminology have gained significant traction [31–36]. Scientific terminology has the potential to perpetuate injustice because the collective use of discipline-specific language helps define how people communicate, sets boundaries around what and how research is conducted and publicized, and indicates communal values. In some cases, the origins of scientific terms are closely tied to a history of oppression (e.g., “slave-making ants”) [37,38]. In other cases, scientific terms can conjure alternate, harmful, meanings that can inadvertently ostracize individuals and groups from the scientific community (e.g., “invasive/alien species,” which echoes xenophobic tropes) [39–42]. Revising disciplinary terminology requires a collective commitment to be more conscientious and intentional about the language we use in science [32]. A growing number of initiatives have participated in this revisionary effort in recent years, including the Better Common Names Project, the Bird Names for Birds initiative, the Gender-Inclusive Biology project, and the Just Language in Ecology Education project. These initiatives highlight the recognition that scientific terminology can negatively impact individuals from marginalized groups.

Among these efforts, the EEB Language Project, a grass-roots initiative first introduced in Cheng and colleagues [32], aims to collate resources and unite EEB scholars in order to identify harmful discipline-specific terms that may warrant reconsideration. To move this effort forward, we launched a large-scale, empirical study to examine EEB community members’ perceptions about discipline-specific terminology and its potential to cause harm. To enact meaningful change in disciplinary culture through the adoption and use of more inclusive language, investigation of the impacts that discipline-specific terminology can have on EEB community members is crucial. To that end, we conducted a mixed-methods study to address the following questions:

1. How do members of the EEB community think that discipline-specific terminology can be harmful, and how do these perceptions vary across demographic groups?
2. How do members of the EEB community perceive they are harmed by discipline-specific terminology, and how do these perceptions vary across demographic groups?
3. What discipline-specific terminology do members of the EEB community identify as harmful?

Through this work, we focus on discipline-specific terminology as an important and accessible leverage-point for creating disciplinary change. In so doing, as an author group we acknowledge that we contribute to a culture of exclusion in EEB through our use of language and aim to rectify this by both calling attention to such patterns and modeling a practice of critical reflection in this manuscript (Box 1). Additionally, we recognize that EEB is an international and multilingual field, and that the terminology discussed in this study primarily reflects English usage in a North American context. While this undoubtedly limits our ability to develop generalizable conclusions and provide recommendations that can be applied across diverse global contexts, we nonetheless view this work as an important first-step toward creating a more inclusive disciplinary environment.

We hope that EEB community members and organizations can leverage our findings to not only identify discipline-specific terminology that may have negative impacts on marginalized communities in our discipline, but to also begin identifying ways that terms could be modified to better support inclusion within EEB and beyond.

**Box 1**

As a group of authors committed to promoting inclusion by critically reflecting on and revising our use of potentially harmful language, we believe it is important to acknowledge that even as we call attention to the potential harms associated with disciplinary terminology in EEB, the language we use in this paper is shaped and constrained by existing disciplinary conventions and our own experiences and limitations as an author team. To provide a few examples: In citations throughout this piece, we use “see” to direct readers to sources; citational convention is one that disability studies scholars have identified as reliant on a sight-related metaphor (e.g., [43]), similar to other disciplinary (dis)ability metaphors we call attention to in our study’s results (see Ability and age section). Throughout much of this article, when discussing disability, we have also chosen to use person-first language (e.g., “a person with disabilities”) as opposed to identity-first (e.g., “a disabled person”) language, both to maintain consistency with the language used in our survey and to honor the fact that respondents tended to use person-first language when discussing their experiences with harmful terminology in EEB. Additionally, to maintain consistency with the language used in our survey, this manuscript uses the suffix “-x” in terms such as “Latinx,” whereas other scholars have recommended alternative gender-inclusive suffixes—such as “-e” in the example “Latine”—to denote gender inclusivity [44].

In short, when calling for greater attention to discipline-specific language, we do so from a stance of humility: We do not purport to use value-free, neutral language—and we include ourselves in the community of those still needing to think carefully and critically about disciplinary terminology and academic conventions. We believe that changing disciplinary norms and language is not something that happens easily, quickly, or all at once; it requires a concerted and sustained communal effort [32,45].

## Methods

To investigate EEB community members’ experiences and perceptions regarding the potential harms of discipline-specific terminology, we conducted a mixed-methods study using an online survey.

### Data collection: Survey development and validity

Our survey consisted of Likert scale and open-ended questions. Survey questions were iteratively revised based on responses from the author team and a pilot with 15 EEB community members. Survey questions asked participants if they thought there was harmful terminology used in EEB and whether they perceived terms used in the discipline to be harmful or offensive to themselves or others. We asked participants who thought there were harmful terms (or were unsure) to share specific examples and explanations, as well as alternative terms that could be effective replacements for the harmful term(s) identified by the participant (see Table 1 for survey prompts). All participants were invited to share demographic information at the end of the survey. Questions about gender, race/ethnicity, and sexual orientation were of a “select all that apply” format. Participants could also opt to self-identify and provide a write-in response that better described their identity. See S1 Material for survey questions used in data analysis. This study was determined to be exempt by the Institutional Review Board at California State University San Marcos (#1861339); participants gave written consent to participate in the study.

**Table 1:** Survey prompts used to evaluate the perceptions EEB community members have about terminology in the discipline that may perpetuate harm.

**Likert scale questions**
I think there is terminology in ecology and evolutionary biology that perpetuates negative stereotypes or impacts individuals or groups negatively.
I have been harmed or offended by terminology used in ecology and evolutionary biology.
**Open response question**
Please list any specific terms (e.g., words or phrases) used in ecology and evolutionary biology that you find harmful or offensive to yourself or others. Fill in as much of the table as you choose.
Please describe in complete, detailed sentences why the terms you listed in the table above are harmful or offensive. Please share as much detail as you feel doing to better help us understand your perspectives.

Participants could respond with “Agree,” “Not sure,” or “Disagree” for each Likert scale question and share multiple terms they identified as harmful.

### Participant recruitment

To identify terms considered harmful by EEB community members, we sought to recruit participants at the graduate level or higher because we believed that increased disciplinary training would enhance their awareness of potentially harmful subfield-specific terms. Thus, participants from the EEB community were recruited in 3 ways. First, we sent email invitations to listservs at non-academic institutions and to biology, ecology, and evolutionary biology departments at various academic institutions (see S1 Table). Second, we advertised the study on social media (e.g., Twitter). Third, we hosted tables at national society conferences in the US that members of the author team attended, including Evolution 2022, the Annual Meeting of the Ecological Society of America 2022, and the Long Term Ecological Research All Scientists Meeting 2022.

The study was administered through a Qualtrics survey during June to December 2022. Study participants were recruited over an extended period to ensure we reached a broad audience and to recruit participants from identities that are systematically excluded from EEB. Participation in the study was voluntary, and participants were not compensated or incentivized to participate.

### Exclusion criteria for collected data

At the end of the 7-month survey period, 2,145 participants responded to the survey. To investigate how various demographic groups in the EEB community perceived the potential impacts of disciplinary terminology, the final data set we analyzed only included participants who provided information for at least 1 demographic question and at least 1 Likert scale or open-ended question asking for examples of terms they considered harmful. Based on the responses that satisfied the above criteria, the final sample size was 795. Because 123 participants did not think there was harmful terminology in the discipline, the sample size for subsequent questions about personal experiences with harmful terminology was 672. Given that participants could opt out of answering any question, sample sizes vary for each analyzed question and demographic group.

If participants selected “Disagree” to the first Likert scale question, survey logic had them skip the second Likert scale and open response questions. We decided this approach was necessary because individuals who do not believe there is harmful terminology are unlikely to contribute harmful terms or discuss their implications. This decision also allowed us to focus on respondents who have experienced or recognized harm from specific terminology, which was the aim of our study. Nearly 15% of individuals in the final data set selected “Disagree” (*n* = 123/795) and thus were not included in subsequent analyses after the first Likert scale question.

### Characterizing participant demographics

To allow for quantitative analysis, we grouped participant responses on gender, race/ethnicity, and sexual orientation into broader demographic categories. Below, we describe how we aggregated participant responses. We note that the use of these broader categories masks the heterogeneity of identities and experiences that exist, and we report disaggregated demographic data in the S1–S6 Figs.

In our analysis, the BIPOC category included participants who selected at least one of the following identities: African American or Black; East Asian; Filipinx or Pacific Islander; Latinx or Hispanic; Native American, American Indian, Alaska Native, Native Hawaiian, or Indigenous Peoples of Canada; Middle Eastern or North African; South Asian; Southeast Asian; wrote-in that they are multiracial; or persons of mixed race who included any of these designations. To examine further possible differences within the BIPOC category, we created 2 subgroups: (1) Persons Excluded due to Ethnicity or Race (PEER; [46]) BIPOC; and (2) Non-PEER BIPOC. PEER BIPOC included participants who self-identified as one of the following: African American or Black; Filipinx or Pacific Islander; Latinx or Hispanic; Native American, American Indian, Alaska Native, Native Hawaiian, or Indigenous Peoples of Canada; Southeast Asian; or participants who included any of these designations. Non-PEER BIPOC included participants who self-identified as East Asian, Middle Eastern or North African, South Asian, or participants who included any of these designations. Those who selected race/ethnicities that fell into both the PEER BIPOC and non-PEER BIPOC categories were only included in the PEER BIPOC category. Participants who identified as only white or who wrote-in that they identified as “multiracial” were not included in either of these categories because they did not specify race/ethnicity (*n* = 5 participants). Although Asian-identifying scholars are still systemically excluded in EEB [47], as are scholars of Middle Eastern and North African descent, we have used the PEER designation [46] as it functions to replace the term “underrepresented minority”. We acknowledge the unique challenges faced by Asian-identifying individuals in EEB [48] and Middle Eastern and North African scholars in EEB, and that their experiences differ from others in the discipline, warranting further examination.

The LGBTQIA+ group included participants who self-identified with at least one of the following: aromantic, asexual, or ace spectrum, bisexual or pansexual or omnisexual, gay, heteroflexible, lesbian, queer, questioning or figuring it out, or any combination of these identities along with straight or heterosexual as participants could select multiple identities.

To examine the experiences of individuals with various gender identities have with terminology in EEB, participants who self-identified as transgender, gender non-conforming, non-binary, genderqueer, genderfluid, agender, Two-Spirit or other Traditional or Indigenous genders, and questioning or figuring it out were classified into a group termed “Trans, Gender Non-Conforming (GNC), Two-Spirit, or Questioning.” Participants who self-identified as a woman, female, and/or feminine were grouped into the “Women or Feminine” group. Participants who self-identified as a man and/or masculine were grouped into “Man or Masculine.” Because participants could identify with multiple genders (*n* = 36/723 participants who shared a gender identity, <5% of participants), some participants were represented in multiple gender categories. For example, a participant who identified as transgender and a woman was grouped into the “Trans, Gender Non-Conforming (GNC), Two-Spirit, or Questioning” group and the “Women or Feminine” group.

Questions about parental education, disability status, immigration, and low-socioeconomic background had 2 response choices (yes/no) that did not require aggregation for analyses.

The demographics for the participants who remained after exclusion criteria were applied can be found in Table 2.

**Table 2:** Summary of participant demographics. Participants could select multiple demographic groups for questions asking which country they grew up in, which country they reside in, their gender identity, sexual orientation, race and ethnicity, field of study, and professional affiliation. Because participants could select more than 1 response for each of these questions, totals for some demographic questions sum to more than 100%. Sample sizes vary by demographic because not all participants responded to each demographic question. Please refer to the Methods section for more information about how participant demographic data were analyzed.

Demographic group	Participants % (*n*)	Demographic group	Participants % (*n*)
***BIPOC*** ^***1***^		***PEER*** ^2^	
BIPOC	25.4% (177)	PEER BIPOC	66.9% (115)
White	74.6% (521)	Non-PEER BIPOC	33.1% (57)
***College education status***		***Immigrant status***	
First-generation	21.2% (160)	Immigrant	18.0% (136)
Continuing generation	78.8% (595)	Not immigrant	82.0% (621)
***Disability status***		***Socioeconomic status***	
Disabled	21.0% (159)	Low-socioeconomic background	26.8% (202)
Not disabled	79.0% (598)	Not low-socioeconomic background	73.2% (553)
***LGBTQIA+ status*** ^3^		***Gender*** ^4^	
LGBTQIA+	34.4% (242)	Transgender, gender non-conforming, two-spirit, or questioning	10.5% (76)
Straight or heterosexual	65.6% (462)	Women or feminine	62.2% (450)
		Men or masculine	32.5% (235)
***Race/ethnicity***		***Sexual orientation*** ^5^	
African American or Black	4.2% (29)	Asexual or ace spectrum	5.3% (37)
East Asian	5.2% (36)	Bisexual or pansexual or omnisexual	15.5% (109)
Filipinx or Pacific Islander	0.9% (6)	Demi-sexual	0.1% (1)
Latinx or Hispanic	9.7% (68)	Gay	5.5% (39)
Middle Eastern or North African	1.6% (11)	Lesbian	3.0% (21)
Multiracial	0.7% (5)	Queer	11.9% (84)
Native American, American Indian, Alaska Native, Native Hawaiian, Indigenous Peoples of Canada	1.9% (13)	Questioning or figuring it out	3.6% (25)
South Asian	2.3% (16)	Straight or heterosexual	67.9% (478)
Southeast Asian	1.1% (8)		
White	82.5% (576)		
***Gender*** ^6^		***Field of study*** ^7^	
Agender	1.5% (11)	Ecology	66.5% (505)
Female	0.3% (2)	Evolutionary biology	44.3% (336)
Feminine	10.0% (72)	Environmental science	19.4% (147)
Man	30.8% (223)	Genetics	14.8% (112)
Masculine	4.0% (29)	Zoology	19.1% (145)
Non-binary	7.6% (55)		
Questioning or figuring it out	2.8% (20)		
Transgender	2.1% (15)		
Two-spirit or other Traditional or Indigenous genders	0.3% (2)		
Woman	59.6% (431)		
***Professional Title***		***Professional affiliation***	
Faculty or lecturer or instructor	50.1% (336)	Academia	90.1% (692)
Graduate student	23.7% (159)	Agency or government	6.8% (52)
Postbacc student	0.7% (5)	Consultant	0.5% (4)
Postdoctoral scholar	12.7% (85)	Industry	2.2% (17)
Research scientist or research associate	6.1% (41)	Museum or aquarium or zoo	2.7% (21)
Staff	3.3% (22)	Non-profit or NGO	3.1% (24)
Undergraduate student	2.2% (15)		
Other	1.2% (8)		
***Countries grew up in*** ^8^		***Country of residence*** ^9^	
United States	77.2% (586)	United States	79.6% (608)
Canada	6.9% (52)	Canada	6.0% (46)
United Kingdom of Great Britain and Northern Ireland	4.1% (31)	United Kingdom of Great Britain and Northern Ireland	3.0% (23)
Germany	1.8% (14)	New Zealand	2.4% (18)
New Zealand	1.6% (12)	Germany	1.3% (10)
Australia	1.3% (10)	Australia	1.2% (9)
Colombia	1.1% (8)	Panama	1.0% (8)
India	1.1% (8)		

^**1**^BIPOC includes participants who self-identified with at least one of the following groups: African American or Black, East Asian; Filipinx or Pacific Islander; Latinx or Hispanic; Native American, American Indian, Alaska Native, Native Hawaiian, or Indigenous Peoples of Canada (e.g., Navajo nation, Blackfeet tribe, Mayan, Aztec, Métis, Inuit, Native Village or Barrow Inupiat Traditional Government, Nome Eskimo Community); Middle Eastern or North African; South Asian; Southeast Asian; self-described themselves as multiracial in the write-in response; or persons of mixed race who included any of these designations.

^2^ PEER (Persons Excluded due to Ethnicity or Race) BIPOC included any participant who self-identified as with at least one of the following groups: African American or Black; Filipinx or Pacific Islander; Latinx or Hispanic; Native American, American Indian, Alaska Native, Native Hawaiian, or Indigenous Peoples of Canada (e.g., Navajo nation, Blackfeet tribe, Mayan, Aztec, Métis, Inuit, Native Village or Barrow Inupiat Traditional Government, Nome Eskimo Community); Southeast Asian; or persons of mixed race who included any of these designations [46]. Non-PEER BIPOC included any participant who self-identified as East Asian, Middle Eastern or North African, South Asian, and did not select one of the groups listed within the PEER BIPOC category. Participants who identified as only white or who wrote in “multiracial” were excluded from the PEER BIPOC and Non-PEER BIPOC designation (*n* = 5 participants).

^3^LGBTQIA+ included participants who selected at least one of the following options: aromantic, asexual, or ace spectrum, bisexual or pansexual or omnisexual, gay, heteroflexible, lesbian, queer, questioning or figuring it out, or any combination of these designations and straight or heterosexual.

^4^Trans, Gender Non-Conforming (GNC), Two-Spirit, or Questioning included individuals who selected one of the following options: transgender, gender non-conforming, non-binary, genderqueer, genderfluid, agender, Two-Spirit or Two-spirit or other Traditional or Indigenous genders, and questioning or figuring it out.

^5^Participants could select multiple sexual orientations. Asexual or Ace spectrum includes participants who identified as Aromantic. Bisexual, pansexual, or omnisexual includes participants who identified as heteroflexible.

^6^Participants could select multiple gender identities. Non-binary includes participants who selected genderfluid, gender non-conforming, and genderqueer or non-binary.

^7^Only the top 5 most frequently selected fields of study are reported above. Participants could select multiple fields of study. The remaining fields (in alphabetical order) along with the percentage of participants who selected those fields are: Agriculture (0.7%), Anatomy (0.1%), Animal behavior/behavioral ecology (2.4%), Biochemistry (0.3%), Biogeochemistry (0.4%), Botany (13.0%), Cell and molecular biology (4.7%), Developmental biology (1.8%), Ecotoxicology (0.1%), Entomology (1.2%), Environmental chemistry (0.1%), Environmental ethics (0.1%), Epidemiology (0.5%), Fisheries (0.3%), Forestry (5.1%), Genetics (14.8%), Genomics (0.3%), Geography (0.1%), Immunology (1.4%), Marine biology (10.7%), Microbiology (6.6%), Mycology (1.4%), Neuroscience (0.3%), Paleontology (0.1%), Physiology (7.5%), STEM education research/Pedagogy (1.6%), Science communication (0.7%), Social science (0.4%), Systems biology (0.1%), Wildlife/Conservation biology (2.6%).

^8^Only countries that were selected by >1% of participants are reported above. Participants could select if they grew up in multiple countries. Remaining participants are from the following countries (in alphabetical order): Afghanistan, Algeria, Andorra, Argentina, Austria, Belarus, Belgium, Botswana, Brazil, Chile, China, Costa Rica, Ecuador, Finland, France, Ghana, Greece, Guyana, Hong Kong (S.A.R.), Ireland, Italy, Jamaica, Kenya, Macedonia, Malaysia, Mexico, Netherlands, Nicaragua, Panama, Peru, Philippines, Poland, Portugal, Puerto Rico, Romania, Russian Federation, Saudi Arabia, Senegal, Serbia, South Africa, South Korea, Spain, Sweden, Switzerland, Taiwan, Thailand, Trinidad and Tobago, Uganda, United Arab Emirates, United Republic of Tanzania, Venezuela, Vietnam, and Zimbabwe.

^9^Only countries that were selected by >1% of participants are reported above. Participants could select if they grew up in multiple countries. Remaining participants are residents of the following countries (in alphabetical order): Afghanistan, Armenia, Austria, Belgium, Brazil, Chile, Costa Rica, Czech Republic, Denmark, Egypt, Estonia, France, Ghana, Hong Kong (S.A.R.), India, Israel, Italy, Netherlands, Norway, Peru, Poland, Republic of Moldova, South Africa, Sweden, Switzerland, Thailand, and Zambia.

### Statistical data analyses

We used multinomial logistic regression to determine if the demographics of participants could predict their responses to questions concerning terminology in EEB. Specifically, we looked at whether participants “Agree,” “Disagree,” or are “Not sure” about whether discipline-specific terms in EEB (1) reinforced negative stereotypes or adversely affected individuals or groups (*n* = 794 participants included in data analysis as one participant from the final data set did not select a response to this question); or (2) have personally harmed the respondent (*n* = 672 participants included in data analysis). Separate multinomial logistic regressions were used to examine whether responses varied by race/ethnicity, parental education level, immigration status, disability status, socioeconomic background, sexual orientation, and gender. For each model, the reference response variable was “Disagree,” and the reference demographic group was the majority-identity in EEB (e.g., “white” for analysis of race/ethnicity). All statistical analyses were conducted in R (v4.2.2) [49]. Multinomial logistic regression models were fit with the *multinom* function in the *nnet* package [50]. Access to the aggregated data and code used to produce the figures are deposited on Dryad [https://doi.org/10.5061/dryad.5x69p8dcv]. Additionally, because our ethics approval prevents us from sharing raw data derived from human participants, we have created simulated data to accompany the code used for the multinomial logistic regressions. Both the simulated data and the statistical analysis code can be accessed on Dryad [https://doi.org/10.5061/dryad.5x69p8dcv].

### Qualitative data analyses

Open-ended survey questions asked participants to list any specific terms (e.g., words or phrases) used in EEB that they perceived as harmful or offensive to themselves or others. We received 817 total responses to this question with 460 unique terms provided. Additionally, participants were prompted to share their rationale for why they thought those terms were harmful. All of the participants’ rationales were de-identified and all participants were assigned pseudonyms for confidentiality. Because some terms differed only in their spelling, punctuation, and suffix, two researchers (A.C.M. and S.J.T.D.) first went through the list of terms and created a list of standardized terms. For example, the standardized term “colonization” grouped together the submitted terms: colonisation, colonization, coloniser, colonizer, colonize, colonizing, colony, and colonial. This standardization process further reduced the 460 unique terms to 227 standardized terms.

The coding of the 227 standardized terms was done in a two-step process involving initial and focused coding [51]. First, 2 researchers (A.C.M. and S.J.T.D) independently reviewed all the terms to create an initial codebook that was approved by all authors. The data set was then coded individually by A.C.M. and S.J.T.D., who met to discuss coding until consensus was reached. Terms were coded to specific themes based primarily on the context provided by the survey participants in their responses. When survey participants did not provide context for identified terms, the analysis team categorized terms based on both their own knowledge and additional research into the meanings of the listed terms. Approximately 1% of participants (*n* = 5/331) shared terms that were not coded because participants did not specify reasons these terms were harmful and we were unable to find EEB scholarship explicitly discussing these terms as sources of harm (e.g., fisherperson, speciose). Terms could be coded under multiple themes unless the submitted term was an eponym, where an eponym may be deemed harmful due to the beliefs of the person the eponym honors. In these cases, the terms were solely coded under the Eponyms theme. Terms were coded to consensus into at least one of 11 themes by A.C.M. and S.J.T.D. (see S2 Table for the codebook).

### Study limitations

Our study has several limitations that warrant consideration. First, we intended to capture a diversity of scientists and professionals across career stages and demographics in our data set; however, most respondents belonged to majority-identity backgrounds. While some of the demographics of our participant pool may more closely mirror the actual demographics of participants from the graduate student level onward that are present in EEB [52,53], we did have a strong representation of some groups whose experiences are often understudied in the field. Our data is unlikely to reflect all of the ways that EEB terminology can harm individuals from marginalized identities, particularly those who were never recruited or retained in EEB. Second, while science is a global enterprise, our respondents (like the authors of this study) were mostly from or living in the United States and Canada, with English as the primary research language. As a result, we do not have a fully global perspective, and we have especially poor representation from Indigenous communities. Third, survey participants were given the option to select “Not sure” as a response to the two Likert scale survey questions, and due to limitations in the survey design we are unable to draw inferences from these responses. Fourth, volunteer bias may be a limitation as individuals who have experienced harm may be more inclined to participate in our survey, potentially inflating our estimates of the proportion of individuals or specific demographic groups who have experienced harm. However, it is crucial to note that these experiences are significant regardless of their prevalence, and this type of bias is unlikely to affect the comparative analyses across different demographic groups. Lastly, survivorship bias may impact our responses as we primarily recruited participants at the graduate level or higher. This may have excluded individuals who left the field earlier in their academic careers, potentially leading to an underrepresentation of perspectives from those who experienced harmful terminology but did not persist in the discipline.

Despite these limitations, we have collected a comprehensive data set that sheds valuable light on the EEB community’s perceptions and experiences regarding harmful discipline-specific terminology.

## Results and discussion

### How do members of the EEB community think that discipline-specific terminology can be harmful, and how do these perceptions vary across demographic groups?

**Fig 1:**
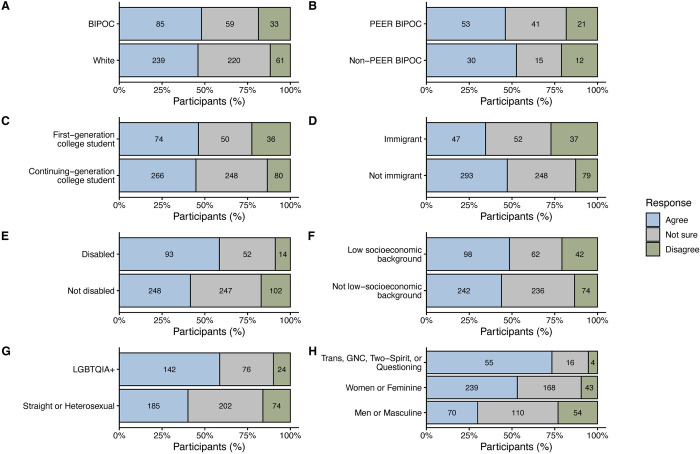
The percent of participants by demographic groups that selected agree (blue), not sure (gray), or disagree (green) that there is terminology in EEB that perpetuates negative stereotypes or impacts individuals or groups negatively. (A) BIPOC included participants that selected one or more of the following: African American or Black; East Asian; Filipinx or Pacific Islander; Latinx or Hispanic; Native American, American Indian, Alaska Native, Native Hawaiian, or Indigenous Peoples of Canada (e.g., Navajo nation, Blackfeet tribe, Mayan, Aztec, Métis, Inuit, Native Village or Barrow Inupiat Traditional Government, Nome Eskimo Community); Middle Eastern or North African; South Asian; Southeast Asian; self-described themselves as multiracial, or persons of mixed race who included any of these designations. (B) PEER [46] BIPOC included any participant that selected at least one of the following: African American or Black; Filipinx or Pacific Islander; Latinx or Hispanic; Native American, American Indian, Alaska Native, Native Hawaiian, or Indigenous Peoples of Canada (e.g., Navajo nation, Blackfeet tribe, Mayan, Aztec, Métis, Inuit, Native Village or Barrow Inupiat Traditional Government, Nome Eskimo Community); Southeast Asian; or persons of mixed race who included any of these designations. Non-PEER BIPOC included any participant that self-identified as East Asian, Middle Eastern or North African, South Asian, or persons of mixed race who included any of these designations. Participants responded to binary response (yes/no) survey questions about whether they identified as (C) the first person in their immediate family to attend college or university, (D) an immigrant, (E) having a disability (either physical or non-physical), or (F) from a low-socioeconomic background. (G) LGBTQIA+ included participants who identified with at least one of the following: aromantic, asexual, or ace spectrum, bisexual or pansexual or omnisexual, gay, heteroflexible, lesbian, queer, questioning or figuring it out, or any combination of these designations and straight or heterosexual. (H) Trans, GNC, Two-Spirit, or Questioning included individuals who identified with at least one of the following: transgender, gender non-conforming, non-binary, genderqueer, genderfluid, agender, Two-Spirit or other Traditional or Indigenous genders, and questioning or figuring it out. Participants could select multiple gender identities. Numbers within the bars represent the number of participants that selected that response. BIPOC, Black, Indigenous, and People of Color; EEB, ecology and evolutionary biology; GNC, gender non-conforming; LGBTQIA+, lesbian, gay, bisexual, transgender, queer/questioning, intersex, and asexual; PEER, Persons Excluded due to Ethnicity or Race.

Overall, 45.5% of participants (*n* = 361/794) agreed that EEB contains terminology that perpetuates negative stereotypes or negatively impacts individuals or groups, while 15.5% disagreed (*n* = 123/794) and 39.0% were not sure (*n* = 310/794). Participants across demographic groups varied in whether they perceived disciplinary terminology as harmful (Fig 1 and Table 3). For example, first-generation college students were 0.6 times less likely to agree that there is harmful terminology in EEB (*p* = 0.045) compared to continuing-generation college students. Immigrants in EEB were 0.3 times less likely to agree that there is terminology in EEB they perceived as harmful relative to participants who identified as non-immigrants (*p* < 0.0001). Individuals with disabilities in EEB had 2.7 times higher odds of agreeing that there is harmful terminology in EEB compared to participants without disabilities (*p* = 0.001). Survey respondents who identified as LGTBQIA+ had 2.4 times higher odds of agreeing that there is harmful terminology in EEB compared to heterosexual individuals (*p* < 0.001). Compared to men or masculine participants, women or feminine participants had 4.3 times higher odds of agreeing there is harmful terminology in EEB (*p* < 0.0001). Even more striking, transgender, gender non-conforming, Two-Spirit, or questioning participants had 10.6 times higher odds of perceiving that there is harmful terminology in EEB relative to men and masculine participants (*p* < 0.0001). Participants who identified as BIPOC, PEER BIPOC participants, and participants from a low-income socioeconomic background were not more likely to agree that there is harmful terminology in EEB relative to their counterparts (*p* > 0.1 for all comparisons).

**Table 3:** Results of multinomial logistic regression predicting which demographic groups report there is terminology in EEB that perpetuates negative stereotypes or impacts individuals or groups negatively. The reference response for the outcome variable is “Disagree” and the reference group for each demographic group is the first group listed in parentheses. Statistical significance (*p* < 0.05) is indicated with bolding. Please refer to the Methods section for information about how participant demographic information was analyzed.

Demographic group (reference group)	Not sure			Agree		
	B (SE)	*p*	Odds ratio	B (SE)	*p*	Odds ratio
BIPOC^1^ (White)	−0.702 (0.261)	**0.00721**	0.496	−0.419 (0.250)	0.0937	0.657
PEER^2^ BIPOC (Non-PEER BIPOC)	0.446 (0.471)	0.344	1.562	0.00949 (0.428)	0.982	1.009
First-generation college student (Continuing generation college student)	−0.803 (0.254)	**0.00155**	0.448	−0.481 (0.240)	**0.0450**	0.618
Immigrant (Not immigrant)	−0.804 (0.251)	**0.00136**	0.448	−1.072 (0.254)	**<0.0001**	0.342
Disabled (Not disabled)	0.428 (0.323)	0.186	1.534	1.005 (0.310)	**0.00118**	2.732
Low socioeconomic (Not low-socioeconomic)	−0.770 (0.240)	**0.00134**	0.463	−0.338 (0.227)	0.137	0.714
LGBTQIA+ (Straight or heterosexual)	0.148 (0.271)	0.548	1.160	0.861 (0.260)	**<0.001**	2.367
Women or feminine (Men or masculine)	0.651(0.238)	**0.00629**	1.918	1.45 (0.245)	**<0.0001**	4.288
Trans, GNC, Two-Spirit, or Questioning (Men or masculine)	0.675 (0.583)	0.247	1.964	2.362 (0.549)	**<0.0001**	10.607

^1^BIPOC refers to Black, Indigenous, and People of Color.

^2^PEER refers to Persons Excluded due to Ethnicity or Race [46].

EEB, ecology and evolutionary biology; GNC, gender non-conforming; LGBTQIA+, lesbian, gay, bisexual, transgender, queer/questioning, intersex, and asexual.

The increased perception of harmful terminology in EEB by individuals with disabilities and those who identify as LGBTQIA+, women or feminine or transgender, gender non-conforming, Two-Spirit, or questioning may in part be informed by the ways that discipline-specific terms in EEB can directly relate to harms historically and currently experienced by members of these groups (e.g., [54–61]). For example, use of the “male/female binary” in EEB risks lending a scientific veneer to anti-trans policies and laws that rely on and reinforce pseudoscientific assumptions about sex and gender binaries [54,62–64]. Similarly, common terms in evolutionary biology, such as “normal” or “defective,” often have eugenic undertones that mirror colloquially used ableist language, harming individuals with disabilities [65–70]. Interestingly, participants who identified as BIPOC, immigrants, or from a low-income socioeconomic background were not more likely to agree that there is harmful terminology in EEB relative to their majority-identity counterparts. While we are unable to speak directly as to why we did not find statistical differences across these groups in our survey, it may be due to the relatively limited representation of these demographic groups within our study. Notably, however, BIPOC participants were more likely than white participants to agree they had been personally harmed by terminology in EEB (see below). These results collectively show that individuals across many marginalized demographic groups do indeed perceive some EEB terminology as harmful or perpetuating negative stereotypes.

### How do members of the EEB community perceive they are harmed by discipline-specific terminology, and how do these perceptions vary across demographic groups?

**Fig 2:**
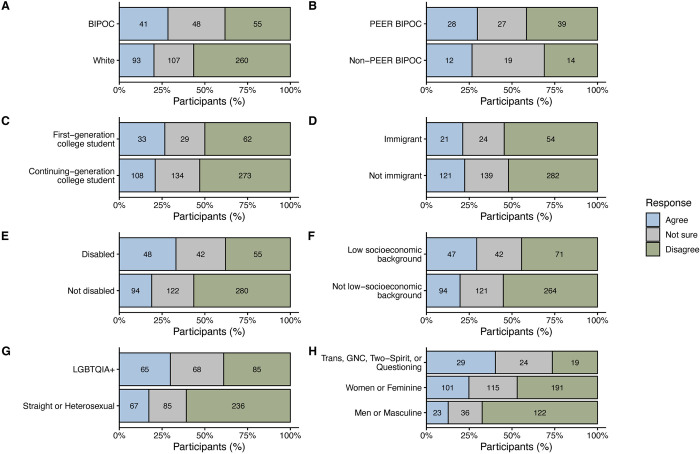
The percent of participants by demographic groups that selected agree (blue), not sure (gray), or disagree (green) that they have been harmed or offended by terminology used in EEB. (A) BIPOC included participants that identified as African American or Black; East Asian; Filipinx or Pacific Islander; Latinx or Hispanic; Native American, American Indian, Alaska Native, Native Hawaiian, or Indigenous Peoples of Canada (e.g., Navajo nation, Blackfeet tribe, Mayan, Aztec, Métis, Inuit, Native Village or Barrow Inupiat Traditional Government, Nome Eskimo Community); Middle Eastern or North African; South Asian; Southeast Asian; self-described themselves as multiracial, or persons of mixed race who included any of these designations. (B) PEER [46] BIPOC included any participant that identified as African American or Black; Filipinx or Pacific Islander; Latinx or Hispanic; Native American, American Indian, Alaska Native, Native Hawaiian, or Indigenous Peoples of Canada (e.g., Navajo nation, Blackfeet tribe, Mayan, Aztec, Métis, Inuit, Native Village or Barrow Inupiat Traditional Government, Nome Eskimo Community); Southeast Asian; or persons of mixed race who included any of these designations. Non-PEER BIPOC included any participant that self-identified as East Asian, Middle Eastern or North African, South Asian, or persons of mixed race who included any of these designations. Participants responded to binary response (yes/no) survey questions about whether they identified as (C) the first person in their immediate family to attend college or university, (D) an immigrant, (E) having a disability (either physical or non-physical), or (F) from a low-socioeconomic background. (G) LGBTQIA+ included participants that identified as aromantic, asexual, or ace spectrum, bisexual or pansexual or omnisexual, gay, heteroflexible, lesbian, queer, questioning or figuring it out, or any combination of these designations and straight or heterosexual. (H) Trans, GNC, Two-Spirit, or Questioning included individuals that identified as transgender, gender non-conforming, non-binary, genderqueer, genderfluid, agender, Two-Spirit or other Traditional or Indigenous genders, and questioning or figuring it out. Participants could select multiple gender identities. Sample sizes within the bars represent the number of participants that indicated that response. BIPOC, Black, Indigenous, and People of Color; EEB, ecology and evolutionary biology; GNC, gender non-conforming; LGBTQIA+, lesbian, gay, bisexual, transgender, queer/questioning, intersex, and asexual; PEER, Persons Excluded due to Ethnicity or Race.

Of the participants who agreed or were not sure about EEB having harmful terminology, 22.2% agreed (*n* = 149/672) that they have been harmed or offended by such terminology, 53.0% disagreed (*n* = 356/672), and 24.9% were not sure (*n* = 167/672). The proportion of participants who agreed, disagreed, or were not sure if they have been harmed by terminology in EEB varied across demographic groups (Fig 2 and Table 4). BIPOC participants in EEB had 2.1 times higher odds of agreeing that they have been harmed by terminology in EEB compared to white participants (*p* = 0.002). However, when we examined these patterns within the PEER BIPOC and non-PEER BIPOC groups, we observed no differences (*p* = 0.838), suggesting consistency in the perception of harmful terminology in EEB across BIPOC groups within our participant population. Individuals with disabilities had 2.6 times higher odds of experiencing harm from disciplinary terminology compared to participants without disabilities (*p* < 0.0001). Participants who identified as coming from a low-socioeconomic background had 1.9 times higher odds of agreeing that they have been harmed by terminology in EEB relative to participants not from a low-socioeconomic background (*p* = 0.005). Members of the LGTBQIA+ community had 2.7 times higher odds of perceiving harm due to terminology in EEB compared to heterosexual participants (*p* < 0.0001). When compared to men or masculine participants, women or feminine participants had 2.8 times higher odds of agreeing they have been harmed by disciplinary terminology (*p* < 0.0001). Compared to men or masculine participants, transgender, gender non-conforming, Two-Spirit, or questioning participants had 8.1 times higher odds of experiencing harm due to EEB terminology (*p* < 0.0001). There were no significant differences in how first-generation college students and continuing-generation college students as well as immigrant and non-immigrant participants perceived harm due to EEB terminology (*p* = 0.223 and *p* = 0.725, respectively).

**Table 4:** Results of multinomial logistic regression predicting which demographic groups report they have been harmed or offended by terminology used in EEB. The reference response for the outcome variable is “Disagree” and the reference group for each demographic group is the first group listed in parentheses. Statistical significance (*p* < 0.05) is indicated with bolding. Please refer to the Methods section for information about how participant demographic information was analyzed.

Demographic groups (reference group)	Not sure			Agree		
	B (SE)	*p*	Odds ratio	B (SE)	*p*	Odds ratio
BIPOC^1^ (White)	0.752 (0.228)	**0.00100**	2.121	0.734 (0.239)	**0.00213**	2.084
PEER^2^ BIPOC (Non-PEER BIPOC)	−0.673 (0.432)	0.119	0.510	−0.177 (0.465)	0.703	0.838
First-generation college student (Continuing generation college student)	−0.0482 (0.248)	0.846	0.953	0.297 (0.244)	0.223	1.345
Immigrant (Not immigrant)	−0.103 (0.266)	0.698	0.902	−0.0983 (0.279)	0.725	0.906
Disabled (Not disabled)	0.561 (0.232)	**0.0155**	1.753	0.955 (0.231)	**<0.0001**	2.600
Low-socioeconomic background (Not low-socioeconomic background)	0.255 (0.223)	0.254	1.291	0.620 (0.223)	**0.00545**	1.859
LGBTQIA+ (Straight or heterosexual)	0.798 (0.206)	**0.0001**	2.221	0.991 (0.215)	**<0.0001**	2.694
Women or feminine (Men or masculine)	0.713 (0.223)	**0.00141**	2.040	1.031 (0.258)	**<0.0001**	2.805
Trans, GNC, Two-Spirit, or Questioning (Men or masculine)	1.454 (0.361)	**<0.0001**	4.281	2.091 (0.373)	**<0.0001**	8.096

^1^BIPOC refers to Black, Indigenous, and People of Color.

^2^PEER refers to Persons Excluded due to Ethnicity or Race [46].

EEB, ecology and evolutionary biology; GNC, gender non-conforming; LGBTQIA+, lesbian, gay, bisexual, transgender, queer/questioning, intersex, and asexual.

With the exception of first-generation college students and immigrant participants, most marginalized groups agreed that they have been harmed by terminology in EEB. These findings may be in part due to the historical and ongoing ways that EEB concepts and terms have participated in systems of violence and oppression (a relationship many participants explicitly referenced in their survey responses, as we describe in the qualitative results section below). For example, concepts and terms from evolutionary biology (e.g., “selection” and “fitness”) have long been wielded to naturalize race and ability hierarchies, supplying pseudoscientific justifications for white supremacy, colonialism, and eugenics [57]. Disciplinary conflations of sex with gender have not only been used to support violent acts against LGBTQIA+ people, but also hinder the understanding of ecological and evolutionary processes [56,71,72]. In classrooms, education research has highlighted how themes of gender essentialism and heteronormativity in biology courses can lower queer students’ sense of belonging and interest in the discipline [73]. Our survey results suggest that there is an urgent need for the discipline to critically reassess its language and adopt practices that foster more inclusive terminology, in line with other educator’s calls for similar work [74,75].

### What discipline-specific terminology do members of the EEB community identify as harmful?

**Fig 3:**
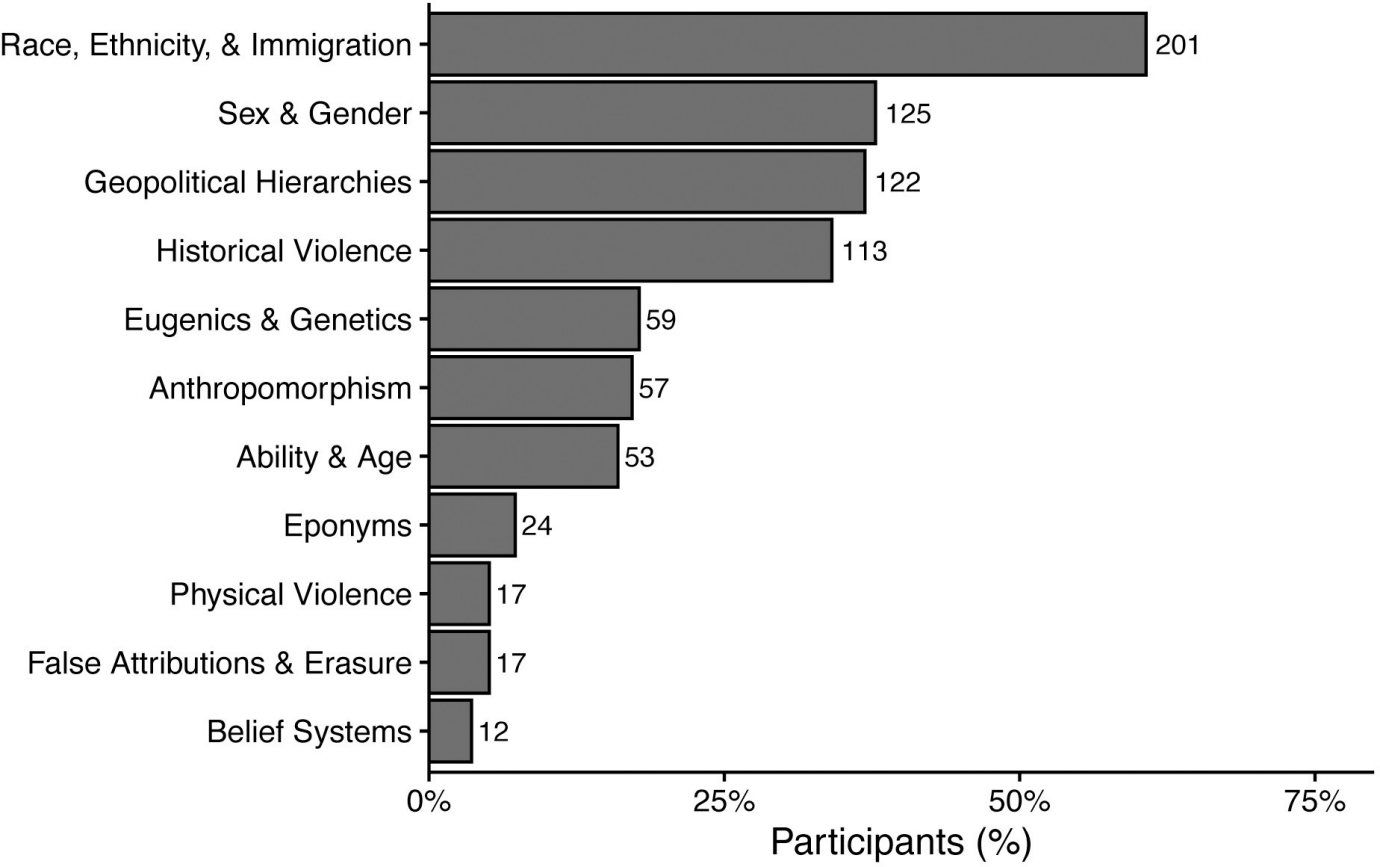
The percent of participants that shared terms that were categorized into eleven emergent themes (*n* = 331 participants). The number of participants that shared terms classified within each theme is listed to the left of the bars. Please note that themes that are less prevalent than others still provide important insights about the exclusionary effects that EEB terminology can have on individuals. EEB, ecology and evolutionary biology.

Participants who reported that they were not sure about or had been harmed or offended by EEB terminology were invited to share terms they perceived as harmful or offensive to themselves and others; there was no limit on the number of terms participants could share. A total of 331 participants shared 227 standardized terms (see Methods for information about term standardization), with each participant sharing a range of 1 to 8 terms. These terms were classified into 11 themes using emergent coding (see S2 Table for the codebook). Below, we detail the 11 themes and provide a qualitative summary along with example terms that were submitted by survey participants. We also provide differential analyses and descriptive statistics for the demographic groups who shared terms more frequently in each theme, if sample size allowed such comparisons. An overview of the prevalence of the 11 themes can be seen in Fig 3. Although quantitative analysis of qualitative data can be helpful for triangulating results, frequency is not necessarily a measure of importance. Terms or themes that are less prevalent can nevertheless provide important insights regarding the exclusionary effects that EEB terminology can have on individuals. It is important to acknowledge that terms considered problematic may not be universally recognized as such by all groups, particularly by those in dominant demographic groups who are less likely to experience the negative impact of these terms. However, when individuals from marginalized groups identify a term as harmful, we consider these sufficient grounds to classify it as problematic and call the EEB community together to move towards a resolution. Our study aims to uplift the voices of those most affected by these terms, as their lived experiences offer critical insights into how discipline-specific terminology can perpetuate exclusion and harm within the field. To amplify the voices of individuals whose identities are systemically excluded in EEB, we have intentionally chosen quotes from these individuals to describe the themes below and have noted the marginalized demographic identities claimed by those participants. Participants quoted have been assigned pseudonyms to maintain their confidentiality.

A comprehensive list of the harmful terms mentioned in the paper, along with suggested alternatives gathered from community members who participated in this study, can be found in S3 Table. We wish to emphasize that this table is not intended to advocate for any singular alternative term. Rather, it presents a summary of harmful terms and plausible alternatives identified by participants to foster a broader conversation within the EEB community. We believe that determining the most appropriate terminology requires a collective, community-driven process and our goal is to facilitate these discussions rather than position ourselves as arbiters of terminology.

#### Race, ethnicity, and immigration

Many participants shared terms that we classified as relating to race, ethnicity, and immigration, including terms related to racist or xenophobic violence, prejudice, or othering. This was the most prevalent theme among participants’ responses, with 60% of participants (*n* = 201/331) sharing 57 standardized terms classified in this theme (Fig 3). More participants who identified as BIPOC shared terms classified in this theme relative to those identifying as white. Sample terms in this theme included “invasive,” “alien,” and “exotic” to describe species moving to a new habitat and the use of “master/slave” to describe interspecies relationships. Any terms that also related to historical violence (e.g., “master/slave”) were additionally coded in the Historical Violence theme (discussed below). Participants reported that terms in this theme can be harmful because they draw on or evoke discourses related to racist, ethnocentric, and nationalist hierarchies and harms (e.g., those related to enslavement and immigration/nativism). For example, Avani, a BIPOC woman who identifies as LGBTQIA+, shared:

“As ecology and evolutionary biology continues to be a white-dominated field, when people talk about invasive species in North America without being aware of or providing context for how invasive species fit into the larger framework of colonialism and racial capitalism in this land, it can feel triggering and offensive especially for recent immigrants of color (especially Asian immigrants that are often stereotyped as foreign and not belonging). This effect is exacerbated [sic] true when militaristic language is associated with invasive species. The rhetoric around the use of ‘invasive’ without care to these larger contexts can contribute to the ranking of ideal individuals and/or genes in relation to their ability to conform, and recreate instances of racism… I am of South Asian descent living in the US and my family has been told to ‘go back to where you came from’ multiple times in different contexts. I cannot ignore the overlaps with invasive species rhetoric and xenophobia nor can I bypass the ecological impacts of the introduced species that are hugely successful in their new ecosystems.”

Avani’s insights highlight the interplay between scientific language and societal perceptions, particularly how terms such as “invasive species” can inadvertently mirror and perpetuate harmful stereotypes and xenophobic attitudes toward immigrants [39–42,76,77]. Nicole, a BIPOC woman and immigrant, further explained the harm these terms can bring to BIPOC individuals:

“The terms exotic and alien are often used to describe people of colour. They’re derogatory terms.”

Because terms like “exotic” or “alien” bring with them a host of harmful associations—particularly for BIPOC individuals as Nicole notes—such terms can send an otherizing message about who is integral (or not) to EEB and to the broader scientific community.

#### Sex and gender

Terms related to sex and gender included those associated with gender identity and/or sexual orientation, terms alluding to misogyny, gendered terms used to describe biological processes, and terms relating to sexualized behavior explicitly related to sexual identity and gender. Any terms that related to sexual violence were only coded in the Physical Violence theme (discussed below). Sex and Gender was the second most prevalent theme, with over one-third of participants (38%, *n* = 125/331) sharing 73 standardized terms categorized in this theme (Fig 3). More participants who identified as transgender, gender non-conforming, Two-Spirit, or questioning shared terms that were coded in this theme relative to participants identifying as women or feminine and men or masculine. The most common terms shared in this theme were “male/female binary,” “gendered terms,” and “hermaphrodite.” Participants considered terms along the “male/female binary” and “gendered terms” harmful and reductive, not only because they essentialized sex and gender, but also because these terms conflated gender with sex—impeding scientific progress in the process [56,78]. For example, moving beyond binary classifications of “sex” in EEB has been found to provide clarifying insights regarding sexual diversity in species, such as white-throated sparrows [71]. Examples like this one can help biologists to confront essentialist misunderstandings about sex and gender that harm marginalized communities and hinder scientific progress [73–75,79]. Terms in this theme can reinforce societal hierarchies and biases against people who identify beyond the gender binary. Carmen, who identified as transgender/genderqueer, LGBTQIA+, from a low-socioeconomic background, and disabled, explained how this kind of harm can occur:

“I’m a nonbinary evolutionary biologist… Describing anatomy, hormones, or chromosomes as inherently ‘male’ or ‘female’ reinforces a bioessentialist view of sex and gender that is actively being used to enact harm on trans communities, and also ignores the complexity of the biological systems at play. Conflation of ‘sex’ and ‘gender’ and the term ‘biological sex’ also contribute to the construction of bioessentialist arguments. Treatment of intermediate sexual phenotypes as ‘abnormal’ or ‘disordered’ is part of a long pattern of pathologizing intersex people, and even when applied to nonhumans, can reinforce that bigotry as well as implying a ‘normal’ condition that may not exist in the system in question. Describing nonhuman organisms as ‘transgender’ (or worse, ‘transvestite’) implies specific social identities, and serves to other trans people as oddities by sensationalizing sexual and gender diversity.”

Carmen is sharing that limiting the description of biological features to a “male” or “female” binary can strengthen arguments used to marginalize people who identify beyond the binary. Recognizing that sex attributes can vary significantly and are phenotypically plastic can help us better understand how ecological and evolutionary systems work, combatting biases that can limit the objectivity of EEB research [80]. Moreover, this reductive binary can frame individuals who are intersex as deviant or deficient—or, in Carmen’s words, “oddities.” In fact, participants identified “hermaphrodite” as a particularly harmful term, noting that it is a slur against intersex people. Anabelle, who identified as transgender/genderqueer, LGBTQIA+, an immigrant, and disabled, further expanded on the dangers of conflating sex and gender:

“Using terms that conflate sex (which nonhuman animals often do have) and gender (which non-animals animals do not have, as far as we have the ability to perceive) can just generally make it harder for transgender and nonbinary identities to be seen as real and for trans/nonbinary individuals to be accepted as valid by those around them. In addition, conflating sex and gender reinforces the idea that sex determines gender, which is a similarly harmful understanding.”

In this way, conflating sex and gender can have marginalizing, exclusionary effects. Scientific terminology can thus participate in naturalizing and normalizing the male/female binary and related gender-biased assumptions [81], lending support to harmful policies that point to “science” and “nature” as pretexts for stigmatizing people who are transgender, gender non-conforming, and Two-Spirit [54,62,64]. Relatedly, participants expressed that scientists should be precise in the language used to contextualize traits and that conflating sex and gender inaccurately describes phenomena, which is a disservice to research [78,80].

#### Geopolitical hierarchies

Terms related to the power and privilege associated with certain geographical regions and place-based belonging (or non-belonging) were placed into the theme Geopolitical Hierarchies. Nearly 37% of participants (*n* = 122/331) shared a total of 18 standardized terms classified in this theme (Fig 3), with more participants identifying as BIPOC sharing terms than those identifying as white. The terms classified in this theme are distinct from the False Attributions and Erasure theme (see below) in that they allude to perceived hierarchies rather than solely the erasure of non-Eurocentric knowledges, including Indigenous Knowledges. Some of the most common terms in the Geopolitical Hierarchies theme included “citizen” and “New World/Old World.” In EEB, “citizen” is often used in the context of “citizen science,” which is scientific work conducted with the participation of the general public (e.g., [82,83]). A longstanding goal of citizen science has been to make science more inclusive by bridging the gap between academic institutions and the general public (e.g., [83–85]). However, there are ongoing scholarly debates about whether this term can have the opposite effect, excluding individuals who are not designated as “citizens” by the government presiding over where they live (e.g., [32,82,86–89]). Maxwell, a BIPOC first-generation college student from a low-socioeconomic background, explains:

“Citizen science in the United States has been a very problematic term. Citizen is a word that has developed a definition revolving around one’s legal status in the country. Many immigrants and/or people that are undocumented are under the impression that they are not welcome to participate in citizen science programs because they are not US citizens. Furthermore, some individuals feel that they would be reported to ICE, police, etc. because they are not citizens. This is particularly the case with Latine members.”

The idea of situating an individual’s engagement in science within a geographic place can inadvertently create a hierarchy where people may not feel comfortable or safe engaging in science. As Maxwell described, people who are not citizens of the country they are living in may be hesitant to engage in public science research, countering one the main goals of “citizen” science [90–93]. While Maxwell’s perspective is based in a US context, survey participants from other countries shared similar perspectives.

In addition, the use of terms like “New World/Old World” to describe the distribution of species in EEB literature and textbooks can reinforce place-based hierarchies. Stefania, a BIPOC woman who is a first-generation college student from a low-socioeconomic background and an immigrant, shared:

“The concept of ‘New’ versus ‘Old’ world is loaded with colonialism and Eurocentrism. The ‘new’ world was new to the Europeans who colonized, but there were people already living in the Americas prior to it. Talking about a ‘new’ or ‘old’ world negates somehow their recognition as inhabitants of the region, negates the idea of colonization and extraction of materials from these regions, and the recognition of people in the ‘new’ areas as humans who contribute to scientific knowledge.”

As Stefania described, these terms do not recognize the Indigenous Peoples who have lived in these regions for thousands of years. These terms discursively erase Indigenous Peoples, framing the “New World” as a region that was not “discovered” until settlers learned about its existence. Participants shared that this Eurocentric framing prioritizes European perspectives and experiences, while ignoring the contributions and existence of Indigenous Peoples. This framing perpetuates persistent “firsting” narratives that are endemic to settler colonialism [94] and emblematic of colonialist science research [95]—narratives that erase and surplant Indigenous perspectives, strategically undermining Indigenous collective continuance [96] (see also [59,97,98]).

#### Historical violence

Terms related to historical violence, colonization, invasion, war, and other forms of discriminatory and eliminationist violence (in the past or ongoing), were classified in the Historical Violence theme. Of the participants who listed harmful terms, 34% (*n* = 113/331) shared terms in this theme for a total of 18 standardized terms (Fig 3). Common terms in this theme include “colonization,” “master/slave,” and “noose,” which have disciplinary-specific uses. For example, the establishment of early successional species on land after a disturbance is often described as “colonization” (by pioneer species, another ode to settler expansion on Indigenous lands). However, “colonization” also describes the historical and ongoing processes of violence, extraction, and domination, whereby settlers seize land and resources through genocidal campaigns against Indigenous communities [96,99]. Many participants shared that applying the term “colonization” to nonhuman animals can normalize and naturalize colonialist violence. Amy, who identified as a woman, LGBTQIA+, an immigrant, and disabled, expressed this sentiment:

“Scientists commonly use the term ‘colonization’ to describe when an organism proliferates in a new (to them) ecosystem, which again invokes European colonialism. Using terms like ‘colonization’ in scientific contexts normalizes them and makes them seem acceptable, thus reducing the amount of power they have in highlighting past and ongoing wrongs still occurring in past/current colonized spaces.”

When used to describe species movement, terms like “colonization” can have the effect not only of normalizing historical harms but also of “mak[ing] them seem acceptable,” hiding the harms of colonization beneath the surface of ostensibly neutral science. Relatedly, participants noted that use of the term “colonization” may be triggering for Indigenous communities, whom settler colonial projects have long targeted and harmed.

In EEB, “master/slave” language is often used both to describe computing resources (e.g., “master” data file or repository) and to describe the behavioral social parasitism in insect species that capture other insect species to increase their number of workers. In entomology, the use of “slave” and other terms related to slavery, caste, and race have been used to describe aspects of social insect biology [100]. In recent years, some scientists have suggested alternative terms to harmful ones used to describe insect behavior (e.g., [37,38]), such as “cleptotectonic” in place of “slave-making” [101]. Even so, these alternatives seem still to be the exception, not the rule. In one open-access literature repository, the terms “master” and “slave” were in over 3,500 publications published in more than 900 life science journals from 2000 to 2020, with the use of these terms increasing each year [35]. Participants also noted that field methods are an area where harmful discipline-specific language can occur. For example, the term “noosing” is used to describe the capturing of lizards in herpetology [35]. Many participants shared that terms like “master/slave” and “noosing” can be triggering for BIPOC, because these terms are steeped in histories of racism [33]—particularly, as participants noted, in the ways these terms relate to abhorrent violence against the Black community in the US. Bethany, a woman, notes:

“Noosing to describe capture of lizards or other animals is absolutely intolerable; any association with common methods of murder and assault are a bad idea, let alone one with such a racialized history.”

In this vein, many participants expressed concerns about how terms describing species interactions and methodologies in EEB, such as “master/slave” and “noosing,” can evoke the history of anti-Black racism and violence. As participants suggested, these terms arguably reduce the historical weight of atrocities, such as enslavement and lynching, and are insensitive to the lived experiences of communities of color—and Black and African American people, specifically.

#### Eugenics and genetics

Terms in the Eugenics and Genetics theme relate to eugenics and associated genetics or phylogenetics concepts that participants deemed harmful, including terms related to developmental hierarchies and to the artificial control of populations and speciation. This theme included 27 standardized terms submitted by 59 participants (18% of all participants; Fig 3). Given that ableist discourse can be fundamentally eugenic in its assumptions and applications, several terms in this theme were also coded in the Ability and Age theme, such as “purity,” “fitness,” and “abnormal,” among others (see [65,66,68,70,102]).

One of the most common terms in the Eugenics and Genetics theme was “primitive,” which is often used in the context of phylogenetics to refer to more ancient or earlier groups. However, “primitive” is too often associated with inferiority and underdevelopment—traits that people in power have long leveraged to justify eugenic efforts to biologically control or eradicate people with disabilities and BIPOC communities, among other minoritized groups (e.g., [103,104]). For example, Armando, who identifies as BIPOC and LGBTQIA+, shared:

“[‘Primitive’] carries a colloquial understanding of ‘less than, less developed, or less evolved’ which mistakenly places the outcome of evolutionary processes on a hierarchy; when discussing species, this leads to the fallacious idea that humans are the pinnacle of evolution. And when further misused by anthropology, it can be weaponized to other non-majority populations or identities and attribute a ‘lower’ or second-class status.”

Using terms like “primitive” to describe traits can invoke discriminatory assumptions about developmental and civilizational hierarchy (e.g., [105,106]). For this reason, in 2007 the Association of Social Anthropologists condemned the use of “primitive” to describe social groups [107]. Along these lines, many participants noted that “primitive” is often used to describe and devalue Indigenous societies, placing them beneath putatively “developed” Western settler colonial cultures. Speaking to a related way that the term “purity” can harbor eugenic connotations and assumptions, Racquel, a BIPOC woman from a low-socioeconomic background and an immigrant, explained:

“As a biracial woman in science I am often informed passively through the language around ‘purity’ and ‘phenotypic preservation’ in this field that my existence is considered anomalous and in some cases just wrong. Many implications around ‘genetic purity’ when discussing preservation of phenotypic traits of species hark back to a time of race science that is still perpetuated by the wrong groups today, this often is at the detriment of the genetic diversity that could be introduced through hybridisation… I find that a lot of our language inadvertently calls to mind that of Nazis and white supremacists. The idea that some things are ‘pure’ and ‘clean’ if they present phenotypically a certain way, whether or not genetic diversity is promoted, can be dangerous.”

Used in this context, terms like “purity” and phenotypic preservation can reinforce the eugenic notion that some phenotypic traits are more desirable than others, and that the mixing of phenotypes—a common phenomenon in nature—is undesirable, even deviant and degenerative [108,109]. For Racquel, these terms have a direct, visceral impact: they send the message that Racquel is impure or “anomalous.” Promoting natural hierarchies premised on phenotypic “purity” not only undermines natural diversity but also echoes eugenic prejudices that underwrite white supremacy [57,108,109]. For Racquel and other participants, such ideas can reinforce barriers to their participation in the field.

#### Anthropomorphism

Anthropomorphism consists of terms that apply human characteristics, behaviors, and relationships to nonhuman entities. This theme included 26 standardized terms from 57 participants (17% of all participants; Fig 3). A majority of this theme’s terms involved projecting human relationships and sexual practices onto nonhuman entities, as was the case with terms like “harem,” “promiscuity,” and “sexy.” Voicing a concern shared by many participants and shared broadly in the scientific community over the past several decades [110,111], Mara, an LGBTQIA+, BIPOC woman, wrote:

“Some terms related to how we discuss sexual selection, behavior, and social/sexual relationships in animals are harmful because they impose heteronormative, sexist, and binary perspectives onto nonhuman animal systems.”

Mara and others highlight how translating societal constructs onto nonhuman animals can be harmful because doing so reinforces and naturalizes prejudices against marginalized groups. These biases may, in turn, shape where and how scientists choose to describe and investigate existing biological phenomena. For example, in ornithology, there has historically been less research on female bird song [24,112] and female avian endocrinology [113], relative to research on these traits in male birds.

Calling attention to the ways anthropomorphism can offer a backdoor for heteronormativity, Hayden, who identifies as BIPOC, LGBTQIA+, disabled, a first-generation college student from a low-socioeconomic background and an immigrant, noted that:

“‘Father/mother’ (and more generally, male/female) in the context in which these terms are casually used but don’t carry adequate context and meaning (most people process these from an anthropocentric stance, and a binary one at that). In most cases, these terms are referring to gamete/offspring production, and in that sense, the term should be explicit: individual[s] can produce a particular gamete type (egg/sperm) and we can describe those roles without ascribing gender-related terms and assumptions. (That is, one can be a father and never donate a gamete; one can donate sperm but be a mother.)”

Hayden’s remarks highlight several important points. First, they call into question whether genetic discussions of sexual reproduction need to adopt language laden with assumptions of heteronormative parental and sexual relationships in humans—particularly when such assumptions are neither accurate nor applicable to biological reproduction, especially in nonhuman animals [74,81,114]. Second, linking parent–child relationships to genetics ignores the possibility of parents who use surrogates or adopt children who do not share their genetic material [75]. And third, defining parental relationships (e.g., “father,” “mother”) in terms of gametes conflates gender with sex—a harmful association described at length in the Sex and Gender theme above.

#### Ability and age

Terms relating to ableism and ageism, including those alluding to normal, optimum, or expected characteristics, were coded in ability and age. This theme included terms from 16% of participants (*n* = 53/331) who shared 32 standardized terms (Fig 3). More participants who identified as disabled or immigrants shared terms in this theme relative to non-disabled and non-immigrant participants. Some of the most common terms in this theme were “fitness” and “blind” or “blindness.” Jackson, who is agender, LGBTQIA+, disabled, and an immigrant, describes some of the impacts these kinds of terms can have:

“As a disabled person, I find that society and sometimes colleagues classify me as ‘low fitness’ based on assumptions around my disability. ‘Survival of the fittest’ is thrown around in both research and the public sphere, especially with COVID, to suggest that people like me deserve to die or should not be prioritized when it comes to life saving treatment based on ‘scientific’ reasoning about my life’s worth.”

Jackson’s experience highlights how terms used in evolutionary biology can perpetuate ableism by associating non-disabled traits with evolutionary advantage and disabled traits with evolutionary disadvantage [57]. In doing so, EEB’s discipline-specific language arguably contributes to the broader social narrative that disabled lives are not as valuable as non-disabled lives, which as Jackson describes, can have real-life consequences, including someone’s access to life-saving healthcare [115,116].

Relatedly, EEB terminology also participates in ableist discourse by repurposing language used to describe cognitive or perceptual differences (e.g., “blindness”). For example, “plant blindness” has been widely used to refer to the broad inability of people to see or notice plants, recognize their role in the environment, and appreciate plant diversity and characteristics—thus those with “plant blindness” treat plants as subordinate to animals and disregard their importance [117–119]. Maybelle, who is an LGBTQIA+ woman and disabled, shares an observation that underscores this issue:

“The term ‘blindness’ is often used to describe a lack of awareness of certain organisms in ecology and evolution, but the term equates the disability condition with a negative connotation that could easily be avoided with a change in the language.”

Maybelle calls attention to the problematic nature of using disability as a metaphor in scientific discourse. By using the term “blindness” to signify unawareness or ignorance in an organism, discipline-specific language assigns a host of negative, deficit connotations to disability [120–122]. As an alternative to this ableist metaphor, the term “plant awareness disparity” has been proposed as an alternative term to explain this phenomenon [123].

Some participants shared how terms in this theme, such as the phrase “survival of the fittest,” have historically been weaponized by scientists and others against individuals who are disabled. Stefan, who is LGBTQIA+ and a first-generation college student, shares:

“Survival of the fittest historically has been used to support eugenics and social Darwinism.”

This phrase has long been wielded beyond its scientific origin to justify eugenics and social Darwinism—ideologies that use discourse about evolution (including “fitness”) as a pretext for imposing prejudicial social hierarchies (see also the Eugenics and Genetics theme above). In this way, “survival of the fittest” has functioned as a rallying cry for exclusionary, discriminatory, and often violent practices and policies aimed at marginalizing or eliminating those deemed “unfit” [57,124].

#### Eponyms

Eponyms refer to the practice of naming entities after a specific individual. Within EEB, eponyms can be found in the scientific and common names of species, award names, and names of scientific phenomena. Nearly 7% of participants (*n* = 24/331, Fig 3) submitted 8 standardized terms that were coded in this theme. Participants described specific eponyms used in EEB as harmful because they honor individuals who advocated for racist, eugenicist, setter-colonial, or otherwise problematic views. Specific examples included “Bachman’s sparrow,” “Townsend’s warbler,” “Fisherian runaway,” and “Cuvier’s beaked whale.” Celeste, a non-binary, LGBTQIA+ woman who is a first-generation college student from a low-socioeconomic background, describes how:

“The horrific legacy of Georges Cuvier and his acts against Sarah Baartman persist today through the goose-beaked whale, a specific animal I study. I have made a push to change the common name of the whale to take away power from Cuvier, a man who was responsible for the origins of the sexualization of Black women.”

Celeste and others are calling attention to the ways that EEB, by naming entities after individuals with violent legacies, tacitly communicates acceptance or indifference toward the harmful perspectives and actions of such figures. Pointing to Cuvier, whose participation in scientific racism is well documented (e.g., [125–128]), Celeste reminds us that through its eponyms, the discipline of EEB not only commemorates Cuvier, it helps his legacy live on. By memorializing figures like Cuvier, the discipline’s eponyms send a problematic message to current and future generations of researchers, testifying to the (questionable) values and principles the EEB community chooses to recognize and celebrate. Eponyms thus can alienate and harm members of the community who are directly impacted by the legacies of these individuals.

Relatedly, many participants expressed broader concerns that “eponyms” were largely problematic in and of themselves: The convention of naming natural entities by branding them with the names of the scientists (often Western and men) who “discovered” them arguably mirrors broader colonial and masculinist practices of claiming and naming the natural world (e.g., [129,130]). Eponyms thus can contribute to the further erasure of Indigenous and minoritized ways of knowing. For example, although 95% of newly described bird species are from the Global South, most of these are named after Western scientists [130]. Given the broad range of issues with eponyms, several naturalists and scholars more broadly have argued against using eponyms in common and species names [131–135].

#### Physical violence

Terms related to physical harm and violence were classified in Physical Violence, with 5% (*n* = 17/331) of participants providing 7 standardized terms (Fig 3). The most common term shared by participants in this theme was “rape,” and other example terms included “attack.” This theme is distinct from Historical Violence, insofar as Physical Violence encompasses acts of violence not specifically linked to particular historical time periods, events, or ideological movements. Many participants stated that terms in this theme are (at least tacitly) misogynistic. Relatedly, the word “rape” to describe animal mating equates these acts with violent human behaviors [136]—a harmful conceptual slippage similar to those discussed in the Anthropomorphism section above. It is important to note that in the early 1980s, the use of the word “rape” was widely challenged, with “forced copulation” becoming the favored alternative term, with few articles since then referring to “rape” in animals [38,136–139]. Vic, who identifies as a woman/genderfluid and LGBTQIA+, responded:

“‘Rape’ is a term that has been used to describe mating systems in, for example, ducks, in which males attempt to forcibly copulate with females. Because the act of rape in human societies is a violent power play, and not a mating strategy, use of the term rape to describe nonhuman behavior is offensive and inappropriate. Its use also promotes the idea that in nonhuman animals, females are passive and unable to make their own decisions, which may carry over to how humans perceive themselves or other humans. Use of the term in EEB is misogynistic.”

This comment points out how equating a mating strategy in nonhuman animals with rape, an act of human-to-human violence, trivializes the severity and gravity of the act; it also helps to naturalize this form of violence. Such comparisons not only misrepresent the nature of the interaction in the animal kingdom, but also undermine the profound psychological and physical trauma experienced by survivors of rape. It also risks retraumatizing survivors of sexual violence through academic and scientific discourse.

#### False attributions and erasure

Terms related to incorrect or inaccurate word choices, often in relation to false attributions, were classified in the False Attributions and Erasure theme. About 5% (*n* = 17/331, Fig 3) of participants submitted 11 standardized terms coded in this theme. Example terms included “pristine” and “discovery/discovered,” which participants noted are often tied to a Eurocentric and Western-centric narrative of “first discoveries” that overlooks and dismisses the role other cultures have had in ecological systems, including Indigenous cultures (see also the Geopolitical Hierarchies theme above). Below, Lexy, an LGBTQIA+ woman, describes how:

“The word ‘discover’ or ‘discovery’ is seen now as a form of erasure of the longstanding, detailed ecological knowledge of Indigenous Peoples’ and cultures.”

Relatedly, other participants shared that the words “discovery” or “discovered” are selectively applied in ways that adopt a Eurocentric perspective on science, treating species or biological phenomena as “discovered” whenever they are first described by Western scientists and publications [140]. This practice can minimize, and even erase Indigenous Knowledges and other marginalized epistemologies, treating Eurocentric contributions to knowledge of the natural world as the whole of science (e.g., [141–143]). Luciana, a BIPOC woman who is a first-generation college student from a low-socioeconomic background and disabled, writes:

“Pristine has colonial origins where Europeans would see the beautiful lab [sic] that was managed by Indigenous people but assumed that Indigenous people had no part in the land management and therefore removed people from the land to be ‘untouched’ and pristine.”

As Luciana notes, describing ecosystems as “pristine” does not give credit to Indigenous communities who have historically cared for ecosystems, often through sophisticated and sustainable practices that have maintained or enhanced biodiversity (e.g., [144–148]). This framing perpetuates the myth of untouched wilderness, erasing evidence of human stewardship that has long shaped these ecosystems [149–152]. By ignoring the contributions of Indigenous peoples, such language reinforces colonial biases and dismisses the value of traditional ecological knowledge.

#### Belief systems

Terminology relating to religion or other personal belief systems, cultural beliefs, and world views were coded in Belief Systems. This theme was least prevalent, with nearly 4% of participants (*n* = 12/331 participants) sharing a total of 8 standardized terms (Fig 3). The terms in this theme fall roughly into 2 categories: (1) species common names with religious associations (e.g., “Wandering Jew,” “Jesus Lizard,” and “Jewfish”); and (2) terms related to religious, spiritual, or cultural beliefs unrelated to EEB (e.g., “Gaia,” “Caste,” “Mother Earth,” and “Design”). Notably, several participants shared that using terms that allude to religion or a designer (e.g., God) to describe common names or biological processes makes them uncomfortable. Julian, who is an LGBTQIA+ woman and identifies as disabled, shared concerns about references to the “Jesus lizard,” writing that:

“Jesus is a holy name for me, and I am not comfortable with its casual use in this context.”

The reptiles in question, “common basilisks” and other members of the genus *Basiliscus*, are sometimes referred to as “Jesus lizards” (or “Jesus Christ lizards”) because they build enough momentum when escaping predators to briefly sprint across bodies of water [153] (see also [154]). Julian’s discomfort highlights a broader ethical concern regarding the use of religious terminology in scientific nomenclature. This practice can be seen by some as disrespectful or irreverent, potentially isolating individuals or communities who hold certain terms sacred.

## Conclusions

In recent years, a growing number of initiatives have called attention to the potential harms of scientific language [31–34]. However, none of these efforts empirically evaluated how members of the scientific community perceive and experience harm due to discipline-specific terminology. To our knowledge, ours is the first study to quantify the prevalence and perceptions of harm associated with terminology in the EEB discipline. We found that a large percentage (45.5%) of survey participants across various demographic groups agreed that EEB contains terminology that perpetuates negative stereotypes or negatively impacts individuals and groups. The similarities in terms provided by participants and the experiences they shared highlight the impacts of the exclusionary history of science, the current barriers to improving representation in EEB, and how harmful discipline-specific terms disproportionately affect those from marginalized groups.

Acknowledging the prevalence and exclusionary potential of harmful terminology in the EEB lexicon is but one move in the direction of the necessary broader change within the discipline. Inclusion can be promoted by reconsidering discipline-specific terminology and replacing harmful terms with alternatives. Indeed, of the 331 participants who submitted harmful terms (see S3 Table), 230 (70%) also provided suggestions for more inclusive alternatives. Individual action is an important mechanism that can support initiatives to reduce harm [32], and our data show that members of the EEB community are already thinking about changes that they can individually make to reduce harm through their intentional choice of language. While we did not formally analyze the alternative terms in our current study, future analysis of these terms may identify additional changes that would contribute to an increased sense of inclusion in EEB. The harmful terms and alternative terms shared by participants are provided in S3 Table, in addition to a public repository hosted by the EEB Language Project, for members of the EEB community to consider as they reflect on, and perhaps revise, their own language choices.

Here, we emphasize the importance of involving the wider EEB community, including perspectives across demographic groups and expertise from sub-disciplines, in the “community-level” work of identifying and discussing alternative terminology [32]. The Entomological Society of America’s Better Common Names Project and the American Ornithological Society’s English Common Names Project, for example, both included community engagement in their processes of re-naming species with known harmful common names [34,155,156]. While many of the common names targeted by these initiatives include eponyms and ethnic slurs, such terms only represent a fraction of those that were identified by participants in our study as harmful. Given that there is no governing body that regulates official usage of much of the discipline-specific terminology in EEB, the process of replacing harmful terms will require broad engagement from members of the EEB community and beyond.

Moving forward, we believe that addressing harmful terminology in EEB must be a community-driven process. We are not advocating for any single alternative term to replace those identified as harmful; rather, we emphasize the importance of collective reflection and discussion across the EEB community. The process of revising and replacing terminology should involve diverse voices and perspectives, ensuring that decisions are made collaboratively [32]. In cases where the community does not reach agreement on alternative terms, education remains a crucial tool for spreading awareness of problematic disciplinary language [30]. Fostering a deeper understanding of the historical and cultural contexts behind specific terms can help the community recognize and redress their adverse impacts, even if immediate consensus on replacement terminology is not achieved. Educating the broader EEB community about the potential harms of terminology can increase awareness and sensitivity within the discipline, leading to more thoughtful language choices.

While terminology is only one aspect of language that perpetuates harm and barriers within EEB, we hope this contribution encourages continued conversations and promotes direct actions toward creating a more inclusive disciplinary culture. To truly achieve cultural change within EEB, there must be continued reflection and revision of terminology at the individual, institutional, and community levels. While this study focused on how language has affected scientists already engaged and working in the EEB discipline, further study is needed to evaluate how language affects the recruitment and retention of individuals pursuing education or careers in EEB. Future research on both the impacts of harmful discipline-specific language and the efficacy of interventions to improve culture through language choices may further inform individual and institutional efforts to build a more inclusive discipline. Only through such disciplinary changes can equitable environments be created where scholars across identities are represented and thrive.

## Supporting information

S1 Material List of survey questions that were used in data analysis. (PDF)

S1 Fig The percent of participants (by race and ethnicity) that selected agree (blue), not sure (gray), or disagree (green) about whether they think there is terminology in EEB that perpetuates negative stereotypes or impacts individuals or groups negatively (*n* = 697 participants). Participants could select more than one race and ethnicity and thus may be represented in multiple bars. Numbers in bars represent the sample size for that demographic group selecting that response.
(PDF)

S2 Fig The percent of participants (by sexual orientation) that selected agree (blue), not sure (gray), or disagree (green) about whether there is terminology in EEB that perpetuates negative stereotypes or impacts individuals or groups negatively (*n* = 703 participants). Participants could select more than one sexual orientation and thus may be represented in multiple bars. Numbers in bars represent the sample size for that demographic group selecting that response.
(PDF)

S3 Fig The percent of participants (by gender) that selected agree (blue), not sure (gray), or disagree (green) about whether there is terminology in EEB that perpetuates negative stereotypes or impacts individuals or groups negatively (*n* = 722 participants). Participants could select more than one gender and thus may be represented in multiple bars. Numbers in bars represent the sample size for that demographic group selecting that response.
(PDF)

S4 Fig The percent of participants (by race and ethnicity) that selected agree (blue), not sure (gray), or disagree (green) that they have been harmed or offended by terminology used in EEB (*n* = 604 participants). Participants could select more than one ethnicity and thus may be represented in multiple bars. Numbers in bars represent the sample size for that demographic group selecting that response.
(PDF)

S5 Fig The percent of participants (by sexual orientation) that selected agree (blue), not sure (gray), or disagree (green) that they have been harmed or offended by terminology used in EEB (*n* = 606 participants). Participants could select more than one sexual orientation and thus may be represented in multiple bars. Numbers in bars represent the sample size for that demographic group selecting that response.
(PDF)

S6 Fig The percent of participants (by gender) that selected agree (blue), not sure (gray), or disagree (green) that they have been harmed or offended by terminology used in EEB (*n* = 622 participants). Participants could select more than one gender and thus may be represented in multiple bars. Numbers in bars represent the sample size for that demographic group selecting that response.
(PDF)

S1 Table Listservs, university departments, and organizations that were invited to participate in the study. (PDF)

S2 Table Codebook used to categorize participants’ submitted examples of harmful terminology in EEB. (PDF)

S3 Table Summary of discipline-specific terms perceived as harmful by study participants that are mentioned in the paper and the suggested alternative terms and/or descriptors shared by study participants. The terms provided here do not reflect all harmful terms participants shared in the study survey. Harmful terms are listed in the order they are discussed in the manuscript. Some harmful terms have been classified under multiple themes. We wish to emphasize that this table is not intended to advocate for any single alternative term. Rather, we present this data to contribute to a broader conversation within the field, recognizing that the appropriate language for the field must be determined through a collective, community-driven process. The authors do not position themselves as the arbiters of these terms but aim to facilitate discussion within the EEB community to consider discipline-specific terms.
(PDF)

## Abbreviations

BIPOC: Black, Indigenous, and People of Color
EEB: ecology and evolutionary biology
GNC: gender non-conforming
LGBTQIA+: lesbian, gay, bisexual, transgender, queer/questioning, intersex, and asexual
PEER: Persons Excluded due to Ethnicity or Race
