# Supplementary material for: Terminology in ecology and evolutionary biology disproportionately harms marginalized groups
Source: PLoS Biol. 2025 Jan 6;23(1):e3002933. doi: 10.1371/journal.pbio.3002933 (PMC11703034; doi:10.1371/journal.pbio.3002933)
Supplement: S1 Material — (PDF) [file pbio.3002933.s001.pdf]

S1 Material. List of survey questions that were used in data analysis.

1. I think there is terminology in ecology and evolutionary biology that perpetuates negative stereotypes or impacts individuals or groups negatively.
    - ○ Agree
    - ○ Disagree
    - ○ Not sure

   Note: if participants selected Disagree, the survey logic had them skip questions 2-4.
2. I have been harmed or offended by terminology used in ecology and evolutionary biology.
    - ○ Agree
    - ○ Disagree
    - ○ Not sure
3. Please list any specific terms (e.g., words or phrases) used in ecology and evolutionary biology that you find harmful or offensive to yourself or others. Fill in as much of the table as you choose.

	Harmful terms	Alternative terms that you have used, seen used, or believe to be effective as replacements for the harmful terms.
1.		
2.		
3.		
4.		
5.		

4. Please describe in complete, detailed sentences why the terms you listed in the table above are harmful or offensive. Please share as much detail as you feel doing to better help us understand your perspectives. (open response)
5. What country(ies) did you grow up in? Select all that apply.

   Note to reader: these are the default options in Qualtrics.
    - ○ Afghanistan
    - ○ Albania
    - ○ Algeria
    - ○ Andorra
    - ○ Angola
    - ○ Antigua and Barbuda
    - ○ Argentina
    - ○ Armenia
    - ○ Australia
    - ○ Austria
    - ○ Azerbaijan
    - ○ Bahamas
    - ○ Bahrain
    - ○ Bangladesh
    - ○ Barbados
    - ○ Belarus
    - ○ Belgium
    - ○ Belize
    - ○ Benin
    - ○ Bhutan
    - ○ Bolivia
    - ○ Bosnia and Herzegovina
    - ○ Botswana
    - ○ Brazil
    - ○ Brunei Darussalam
    - ○ Bulgaria
    - ○ Burkina Faso
    - ○ Burundi
    - ○ Cambodia
    - ○ Cameroon

- Canada
- Cape Verde
- Central African Republic
- Chad
- Chile
- China
- Colombia
- Comoros
- Costa Rica
- Côte d'Ivoire
- Croatia
- Cuba
- Cyprus
- Czech Republic
- Democratic Republic of the Congo
- Denmark
- Djibouti
- Dominica
- Dominican Republic
- Ecuador
- Egypt
- El Salvador
- Equatorial Guinea
- Eritrea
- Estonia
- Ethiopia
- Fiji
- Finland
- France
- Gabon
- Gambia
- Georgia
- Germany
- Ghana
- Greece
- Grenada
- Guatemala
- Guinea
- Guinea-Bissau
- Guyana
- Haiti
- Honduras
- Hong Kong (S.A.R.)
- Hungary
- Iceland
- India
- Indonesia
- Iran
- Iraq
- Ireland
- Israel
- Italy
- Jamaica
- Japan
- Jordan
- Kazakhstan
- Kenya
- Kiribati
- Kuwait
- Kyrgyzstan
- Lao People's Democratic Republic
- Latvia
- Lebanon
- Lesotho
- Liberia
- Libyan Arab Jamahiriya
- Liechtenstein
- Lithuania
- Luxembourg
- Madagascar
- Malawi
- Malaysia
- Maldives
- Mali
- Malta
- Marshall Islands
- Mauritania
- Mauritius
- Mexico
- Micronesia
- Monaco
- Mongolia
- Montenegro
- Morocco
- Mozambique
- Myanmar
- Namibia
- Nauru
- Nepal
- Netherlands
- New Zealand
- Nicaragua
- Niger
- Nigeria
- North Korea
- Norway
- Oman
- Pakistan
- Palau
- Panama
- Papua New Guinea
- Paraguay
- Peru
- Philippines
- Poland
- Portugal
- Qatar
- Republic of Moldova
- Romania
- Russian Federation
- Rwanda
- Saint Kitts and Nevis
- Saint Lucia
- Saint Vincent and the Grenadines
- Samoa
- San Marino
- Sao Tome and Principe
- Saudi Arabia
- Senegal
- Serbia
- Seychelles

- Sierra Leone
- Singapore
- Slovakia
- Slovenia
- Solomon Islands
- Somalia
- South Africa
- South Korea
- Spain
- Sri Lanka
- Sudan
- Suriname
- Swaziland
- Sweden
- Switzerland
- Syrian Arab Republic
- Tajikistan
- Thailand
- The former Yugoslav Republic of Macedonia
- Timor-Leste
- Togo
- Tonga
- Trinidad and Tobago
- Tunisia
- Turkey
- Turkmenistan
- Tuvalu
- Uganda
- Ukraine
- United Arab Emirates
- United Kingdom of Great Britain and Northern Ireland
- United Republic of Tanzania
- United States of America
- Uruguay
- Uzbekistan
- Vanuatu
- Venezuela
- Vietnam
- Yemen
- Zambia
- Zimbabwe

6. What is your country of current residence? Select all that apply.
    - Same options as previous question
7. What is your current field of study? Select all that apply.
    - Botany
    - Cell and molecular biology
    - Developmental biology
    - Ecology
    - Environmental science
    - Evolutionary biology
    - Forestry
    - Genetics
    - Immunology
    - Marine biology
    - Microbiology
    - Mycology
    - Physiology
    - Zoology
    - Prefer to write in: (open response)
8. What is your current professional affiliation?
    - Academia
    - Agency or Government
    - Industry
    - Museum or Aquarium or Zoo
    - Non-profit or NGO
    - Prefer to write in: (open response)

    If Academia, prompted to this question:

i. What is your current professional title?
   1. Undergraduate student
   2. Postbacc student
   3. Graduate student
   4. Postdoctoral scholar
   5. Research scientist or research associate
   6. Faculty or lecturer or instructor
   7. Staff
   8. Prefer to write in: (open response)

9. Please indicate the identity(ies) you feel most closely describe your race(s) and ethnicity(ies). Select all that apply.
   - African American or Black
   - Filipinx or Pacific Islander
   - Latinx or Hispanic
   - East Asian
   - South Asian
   - Southeast Asian
   - Middle Eastern or North African
   - Native American, American Indian, Alaska Native, Native Hawaiian, Indigenous Peoples of Canada (e.g.: Navajo nation, Blackfeet tribe, Mayan, Aztec, Métis, Inuit, Native Village or Barrow Inupiat Traditional Government, Nome Eskimo Community, etc.)
   - White
   - Decline to state
   - Prefer to self-identify or elaborate my selection(s): (open response)
10. Please indicate the identity(ies) you feel most closely describe your gender(s). Select all that apply.
   - Agender
   - Feminine
   - Woman
   - Genderfluid
   - Genderqueer or Non-binary
   - Gender non-conforming
   - Intersex
   - Masculine
   - Man
   - Questioning or figuring it out
   - Transgender
   - Two-spirit or other Traditional or Indigenous genders
   - Decline to state
   - Prefer to self-identify or elaborate my selection(s): (open response)
11. Please indicate the identity(ies) you feel most closely describe your sexual orientation(s). Select all that apply.
   - Asexual or Ace spectrum
   - Bisexual or Pansexual or Omnisexual
   - Gay

- Lesbian
- Straight or Heterosexual
- Questioning or figuring it out
- Queer
- Decline to state
- Prefer to self-identify or elaborate my selection(s): (open response)

12. Are you the first person in your immediate family to attend college or university?
    - No
    - Yes
13. Do you come from a low socioeconomic background?
    - No
    - Yes
14. Are you an immigrant?
    - No
    - Yes
15. Do you have a disability (either physical or non-physical)?
    - No
    - Yes
