# Supplementary material for: Terminology in ecology and evolutionary biology disproportionately harms marginalized groups
Source: PLoS Biol. 2025 Jan 6;23(1):e3002933. doi: 10.1371/journal.pbio.3002933 (PMC11703034; doi:10.1371/journal.pbio.3002933)
Supplement: S2 Fig — Participants could select more than one sexual orientation and thus may be represented in multiple bars. Numbers in bars represent the sample size for that demographic group selecting that response. (PDF) [file pbio.3002933.s003.pdf]

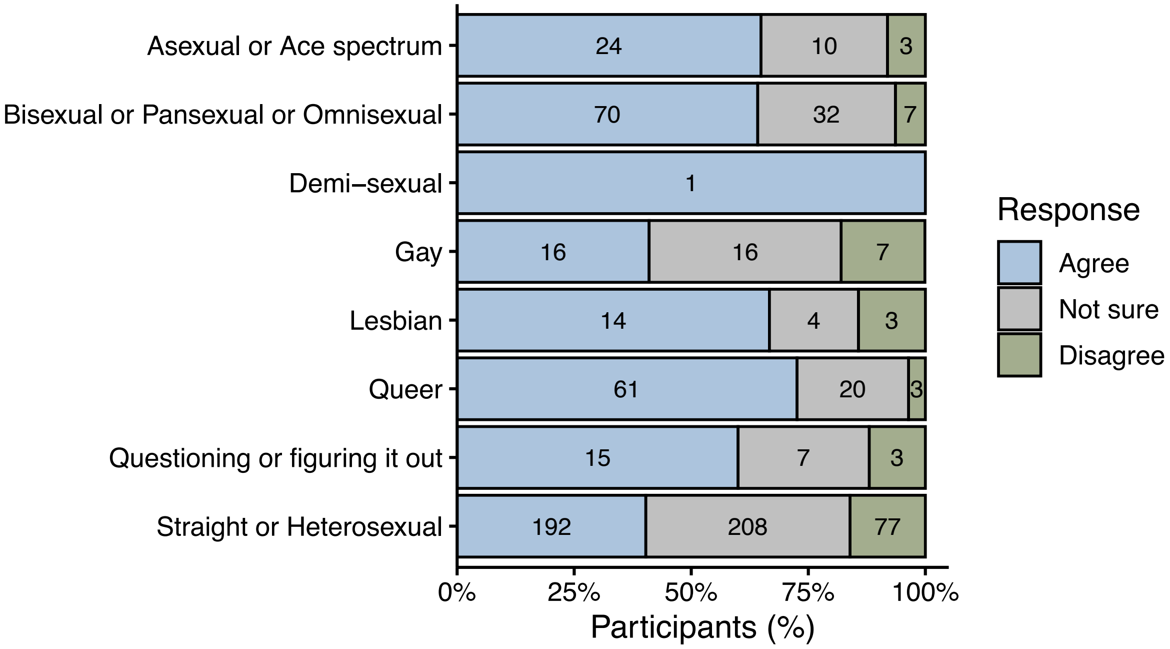

S2 Fig. The percent of participants (by sexual orientation) that selected agree (blue), not sure (grey), or disagree (green) about whether there is terminology in EEB that perpetuates negative stereotypes or impacts individuals or groups negatively (*n* = 703 participants). Participants could select more than one sexual orientation and thus may be represented in multiple bars. Numbers in bars represent the sample size for that demographic group selecting that response.
