# Supplementary material for: Terminology in ecology and evolutionary biology disproportionately harms marginalized groups
Source: PLoS Biol. 2025 Jan 6;23(1):e3002933. doi: 10.1371/journal.pbio.3002933 (PMC11703034; doi:10.1371/journal.pbio.3002933)
Supplement: S6 Fig — Participants could select more than one gender and thus may be represented in multiple bars. Numbers in bars represent the sample size for that demographic group selecting that response. (PDF) [file pbio.3002933.s007.pdf]

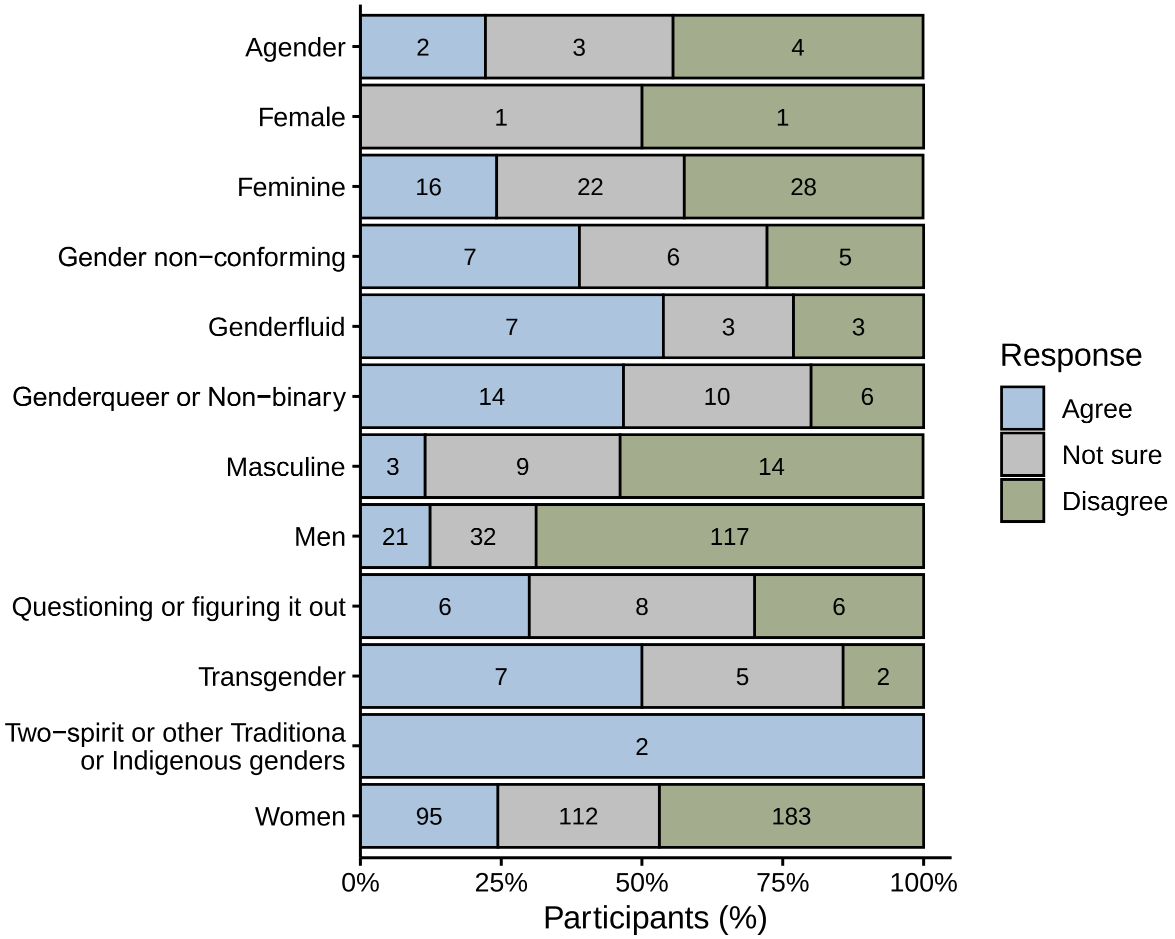

S6 Fig. The percent of participants (by gender) that selected agree (blue), not sure (grey), or disagree (green) that they have been harmed or offended by terminology used in EEB (*n* = 622 participants). Participants could select more than one gender and thus may be represented in multiple bars. Numbers in bars represent the sample size for that demographic group selecting that response.
