# Supplementary material for: Terminology in ecology and evolutionary biology disproportionately harms marginalized groups
Source: PLoS Biol. 2025 Jan 6;23(1):e3002933. doi: 10.1371/journal.pbio.3002933 (PMC11703034; doi:10.1371/journal.pbio.3002933)
Supplement: S1 Table — (PDF) [file pbio.3002933.s008.pdf]

S1 Table. Listservs, university departments, and organizations that were invited to participate in the study.

**Listservs, University Departments, and Organizations**
Boston University Biogeoscience Program (https://www.bu.edu/bio-geo/)
Boston University Department of Biology (https://www.bu.edu/biology/)
Bowling Green State University Department of Biological Sciences (https://www.bgsu.edu/arts-and-sciences/biological-sciences.html)
California State University Northridge Department of Biology (https://www.csun.edu/science-mathematics/biology)
Club Eco-Evo Latinoamerica (https://ecoevolat.github.io/)
Columbia University Department of Ecology, Evolution and Environmental Biology (https://e3b.columbia.edu/)
Diversify EEB (https://diversifyeeb.com/)
EcoED List (https://groups.google.com/g/ecoed)
ECOLOG-L (https://www.esa.org/membership/ecolog/)
Ecological Society of America: Black Ecologists Section (https://www.esa.org/blackecologists/)
Ecological Society of America: Environmental Justice Section (https://www.esa.org/enjustice/)
Ecological Society of America: Inclusive Ecology Section (https://www.esa.org/inclusive-ecology/)
Ecological Society of America: Latin American & Caribbean Chapter (https://www.esa.org/latinamerican/)
GeoLatinas (https://geolatinas.org/)
Graduate Student and Postdoctoral SACNAS Chapter at University of California, Davis (https://gspdsacnasatucd.weebly.com/)
Harvard Department of Organismic and Evolutionary Biology (https://oeb.harvard.edu/)
Harvard Forest (https://harvardforest.fas.harvard.edu/)
Harvard Forest Summer Research Program in Ecology (https://harvardforest.fas.harvard.edu/other-tags/reu)
Inclusive Scicomm (https://inclusivescicomm.org/)
Indiana University Bloomington Department of Biology (https://biology.indiana.edu/index.html)
Long-Term Ecological Research Network (https://lternet.edu/)
National Center for Ecological Analysis and Synthesis (https://www.nceas.ucsb.edu/)
Penn State Department of Biology (https://science.psu.edu/bio)
Penn State Ecology Program (https://www.huck.psu.edu/graduate-programs/ecology)
Portland State University, Department of Environmental Science & Management (https://www.pdx.edu/environmental-science/)
Princeton University Department of Ecology and Evolutionary BIology (https://eeb.princeton.edu/)

Rutgers University, Newark, Department of Earth and Environmental Sciences (https://sasn.rutgers.edu/earth-and-environmental-sciences)
Smithsonian Tropical Research Institute IDEA (Inclusion, Diversity, Equity and Access) Group (https://www.stri-idea.org/)
Society for Integrative and Comparative Biology (https://sicb.org/)
Society for the Advancement of Biology Education Research (https://saberbio.wildapricot.org/)
Stanford Doerr School of Sustainability (https://sustainability.stanford.edu/)
Rutgers University, New Brunswick, The School of Environmental and Biological Sciences (https://sebs.rutgers.edu/)
Undergraduate Field Experiences in Research Network (https://ufern.net/)
University of California, Berkeley Department of Environmental Science, Policy, and Management (https://ourenvironment.berkeley.edu/)
University of California, Berkeley Department of Plant and Molecular Biology (https://plantandmicrobiology.berkeley.edu/)
University of California, Berkeley Integrative Biology (https://ib.berkeley.edu/)
University of California, Davis Animal Behavior Graduate Group (https://grad.ucdavis.edu/programs/ganb)
University of California, Davis Center for Population Biology (https://cpb.ucdavis.edu/)
University of California, Davis Department of Evolution and Ecology (https://eve.ucdavis.edu/)
University of California, Davis Department of Wildlife, Fish, and Conservation Biology (https://wfcb.ucdavis.edu/)
University of California, Davis Graduate Group in Ecology (https://ecology.ucdavis.edu/)
University of Georgia, Warnell School of Forestry and Natural Resources Faculty (https://warnell.uga.edu/)
University of Michigan Department of Ecology and Evolutionary Biology (https://lsa.umich.edu/eeb)
University of Wisconsin Department of Integrative Biology (https://integrativebiology.wisc.edu/)
Women of Color & Non-Binary People of Color in Ecology & Evolutionary Biology Slack Group (https://www.wocineeb.org/index.html)
Yale University Department of Ecology and Evolutionary Biology (https://eeb.yale.edu/)
Yale University School of the Environment (https://environment.yale.edu/)
