# Supplementary material for: Terminology in ecology and evolutionary biology disproportionately harms marginalized groups
Source: PLoS Biol. 2025 Jan 6;23(1):e3002933. doi: 10.1371/journal.pbio.3002933 (PMC11703034; doi:10.1371/journal.pbio.3002933)
Supplement: S2 Table — (PDF) [file pbio.3002933.s009.pdf]

S2 Table. Codebook used to categorize participants' submitted examples of harmful terminology in EEB.

Category	Characteristics
**Race, Ethnicity, & Immigration**	Terms relating to race, ethnicity, and all forms of xenophobia and associated prejudices. Terms eluding to actions and violence based on race, ethnicity, and xenophobia. Terms alluding to the "othering" of phenomena/organisms based on race, ethnicity, and xenophobia. Terms relating to human-centric hierarchies and actions and/or violence based on beliefs of race, ethnicity, and xenophobia.
**Sex & Gender**	Terms relating to sex and gender identity and sexual orientation, terms alluding to misogyny, gendered terms used to describe biological phenomena, and terms relating to sexualized behavior explicitly tied to sexual identity and gender. Terms related only to sexual violence were only coded under "Physical Violence."
**Geopolitical Hierarchies**	Terms relating to perceived geographical or geopolitical hierarchies. Terms relating to place-based belonging (or not belonging). Terms that denigrate based on location.
**Historical Violence**	Terms relating to historical violence, colonization, invasion, war, and all forms of violence targeting specific groups both throughout history and presently.
**Eugenics & Genetics**	Terms relating to eugenics and other ideas regarding genetics or phylogenetics that are deemed harmful. Terms related to the artificial control of populations and speciation.
**Anthropomorphism**	Applying human-centric behaviors and ideas to non-human entities.
**Ability & Age**	Terms relating to ableism and ageism, including terms that allude to normal/optimum/expected characteristics, and lead to an othering of all characteristics not deemed to be the expected norm.
**Eponyms**	Eponyms have all been included in this theme. While the basis for an eponym being considered harmful could result in cross-listing with another theme, based on the context regarding the inappropriate context of the eponym (i.e., the harmful context behind the eponym could be in relation to Sex & Gender; Race, Ethnicity, & Immigration; Eugenics & Genetics; Ability & Age, etc.), we have decided to prioritize coding eponyms in this category and have not cross-listed them into multiple categories.
**Physical Violence**	Terms relating to physical harm and violence.
**False Attributions & Erasure**	Terms relating to incorrect/inaccurate word choices, often used to describe false attributions.
**Belief System**	Terms relating to religion or other personal or cultural belief systems and driving world views.
