# Supplementary material for: Terminology in ecology and evolutionary biology disproportionately harms marginalized groups
Source: PLoS Biol. 2025 Jan 6;23(1):e3002933. doi: 10.1371/journal.pbio.3002933 (PMC11703034; doi:10.1371/journal.pbio.3002933)
Supplement: S3 Table — The terms provided here do not reflect all harmful terms participants shared in the study survey. Harmful terms are listed in the order they are discussed in the manuscript. Some harmful terms have been classified under multiple themes. We wish to emphasize that this table is not intended to advocate for any single alternative term. Rather, we present this data to contribute to a broader conversation within the field, recognizing that the appropriate language for the field must be determined through a collective, community-driven process. The authors do not position themselves as the arbiters of these terms but aim to facilitate discussion within the EEB community to consider discipline-specific terms. (PDF) [file pbio.3002933.s010.pdf]

S3 Table. Summary of discipline-specific terms perceived as harmful by study participants that are mentioned in the paper and the suggested alternative terms and/or descriptors shared by study participants. The terms provided here do not reflect all harmful terms participants shared in the study survey. Harmful terms are listed in the order they are discussed in the manuscript. Some harmful terms have been classified under multiple themes. We wish to emphasize that this table is not intended to advocate for any single alternative term. Rather, we present this data to contribute to a broader conversation within the field, recognizing that the appropriate language for the field must be determined through a collective, community-driven process. The authors do not position themselves as the arbiters of these terms but aim to facilitate discussion within the EEB community to consider discipline-specific terms.

Harmful Term Shared by Study Participants	Alternative Term(s) and/or Descriptors Shared by Study Participants	Themes
Invasive	Non-Indigenous species, Non-endemic, Newly arrived species, Nuisance species, Problem species, Opportunistic species	Race, Ethnicity, & Immigration<br>Historical Violence
Alien		Race, Ethnicity, & Immigration<br>Geopolitical Hierarchies
Exotic		Race, Ethnicity, & Immigration<br>Geopolitical Hierarchies
Male / Female Binary	Use specific terms that describe the biological sex of the organism (e.g., male or female) rather than conflating gender and biological sex. Describe the trait being discussed (e.g., anatomical descriptors, individuals with testes / ovaries, egg / sperm producing organism, staminate/pistillate flowers, monoecious individuals, XY individual, hormonal descriptors).	Sex & Gender
Gendered Terms (e.g., gender for non-human organisms, gender differences)*		Sex & Gender
Hermaphrodite	Intersex, Monoecious, Description of specific reproductive systems	Sex & Gender
Citizen	Community science, Participatory science, Volunteer science	Race, Ethnicity, & Immigration<br>Geopolitical Hierarchies
New World / Old World	Specify the location, Africa & Asia / Americas & Europe, Western / Eastern Hemisphere	Geopolitical Hierarchies<br>False Attributions & Erasure
Colonization / Colonizer	Establishment, spread, or expansion of a population, Early successional species	Geopolitical Hierarchies<br>Historical Violence
Master / Slave	Cleptotectonic, Leistic behavior, Host / parasite, Brood parasite, "Pirate" ants	Race, Ethnicity, & Immigration<br>Historical Violence
Noose	Lasso	Race, Ethnicity, & Immigration<br>Historical Violence
Primitive	Ancestral	Race, Ethnicity, & Immigration<br>Geopolitical Hierarchies
Purity	Parental species, non-admixed, homogenous	Race, Ethnicity, & Immigration<br>Eugenics & Genetics<br>Ability & Age
Harem	Mating group, single male troop, pair bonded mate group	Sex & Gender<br>Anthropomorphism
Promiscuity	Multiple mating system	Anthropomorphism
Sexy	Preferred	Anthropomorphism
Father / Mother	Parent gamete, egg / sperm	Sex & Gender<br>Anthropomorphism

Fitness / Survival of the Fittest	Adapted, natural selection	Eugenics & Genetics<br>Ability & Age
Blind / Plant Blindness	Masked, Plant awareness disparity	Ability & Age
Eponyms (e.g., Bachman's sparrow, Townsend's warbler, Fisherian runaway, Cuvier's beaked whale)*	When naming organisms, use common names that describe the appearance of organisms and/or use the Latin name. Remove the names of problematic scientists from biological processes.	Eponyms
Rape	Forced copulation, Coerced copulation	Anthropomorphism<br>Physical Violence
Discovery / Discovered	Identified or described, known to Western science	False Attributions & Erasure
Pristine	Indigenous ecosystem	False Attributions & Erasure
Species names with religious associations (e.g., Wandering Jew, Jesus Lizard, Jewfish)*	When naming organisms, use common names that describe the appearance of organisms and/or use the Latin name.	Belief Systems<br><br>*Some terms also coded in*<br>Race, Ethnicity, & Xenophobia
Terms related to religious, spiritual, or cultural beliefs (e.g., Gaia, Caste, Mother Earth, Design)*	Use specific terms to describe ecosystems and ecosystem processes (e.g., stochasticity to describe variability). Use specific terms to describe an organism's behavior or role in an ecosystem.	Belief Systems<br><br>*Some terms also coded in*<br>Race, Ethnicity, & Xenophobia<br>Sex & Gender<br>Historical Violence

* Indicates that the listed term represents a broader grouping rather than a specific term shared by study participants. For example, the entry "Gendered Terms" refers to a grouping encompassing various terms, some of which were shared by study participants and are mentioned in the text (e.g., gender for non-human organisms, gender differences). Where applicable, examples of specific terms that fall within these broader themes are provided in the table.
